# Design, synthesis, and physicochemical study of a biomass-derived CO_2_ sorbent 2,5-furan-bis(iminoguanidine)

**DOI:** 10.1016/j.isci.2021.102263

**Published:** 2021-03-04

**Authors:** Qianzhong Zhang, Yi Jiang, Yinwu Li, Xianheng Song, Xiang Luo, Zhuofeng Ke, Yong Zou

**Affiliations:** 1 School of Pharmaceutical Sciences, Sun Yat-sen University, Guangzhou 510000, P. R. China; 2 School of Chemistry, Sun Yat-sen University, Guangzhou 510000, P. R. China; 3 Guangdong Provincial Key Laboratory of Chiral Molecule and Drug Discovery, Guangzhou 510000, P. R. China

**Keywords:** Chemistry, Organic Synthesis, Natural Product Chemistry, Environmental Science, Pollution, Biomaterials

## Abstract

In this study, the concept of biomass-based direct air capture is proposed, and the aminoguanidine CO_2_ chemical sorbent 2,5-furan-bis(iminoguanidine) (FuBIG) was designed, synthesized, and elucidated for the physicochemical properties in the process of CO_2_ capture and release. Results showed that the aqueous solution of FuBIG could readily capture CO_2_ from ambient air and provided an insoluble tetrahydrated carbonate salt FuBIGH_2_(CO_3_) (H_2_O)_4_ with a second order kinetics. Hydrogen binding modes of iminoguanidine cations with carbonate ions and water were identified by single-crystal X-ray diffraction analysis. Equilibrium constant (K) and the enthalpies (ΔH) for CO_2_ absorption/release were obtained by thermodynamic and kinetic analysis (K_7_ = 5.97 × 10^4^, ΔH_7_ = −116.1 kJ/mol, ΔH_8_ = 209.31 kJ/mol), and the CO_2_-release process was conformed to the geometrical phase-boundary model (1-(1-α)^1/3^ = kt). It was found that the FuBIGH_2_(CO_3_) (H_2_O)_4_ can release CO_2_ spontaneously in DMSO without heating. Zebrafish models revealed a favorable biocompatibility of FuBIG.

## Highlights

• The concept of biomass-derived direct air capture has been proposed and reinforced by the sorbent of FuBIG
• The sorbent revealed favorable thermodynamic and kinetic parameters for CO_2_ capture and release
• The sorbent has excellent biocompatibility and good thermostability
• FuBIG carbonate can release CO_2_ and regenerate FuBIG spontaneously in DMSO without heating

## Graphical abstract

**Figure:**
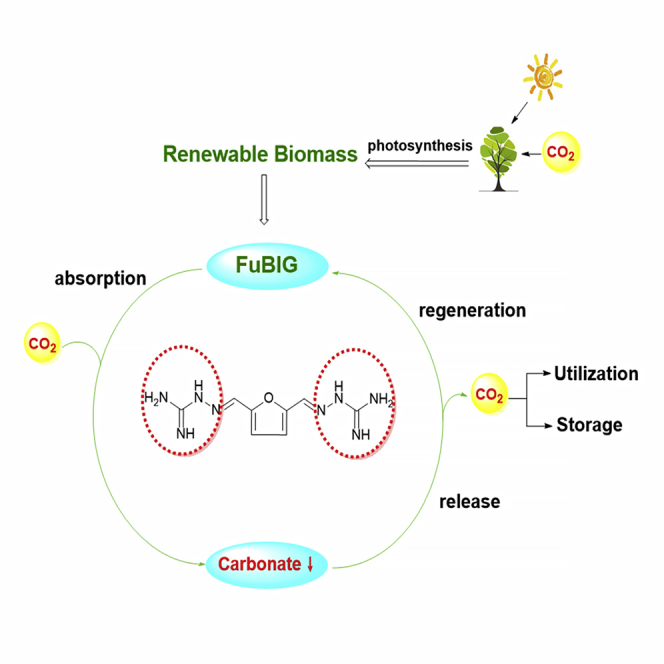

## Introduction

The heavy reliance and massive consumption of fossil resources in modern society has caused continuous rising of atmospheric CO_2_ concentration and resulted in an alarming change of global climate (Cox et al., 2020; Davis et al., 2018; Anderson et al., 2018; Jin et al., 2020). Carbon capture and storage (CCS) has been proposed and implemented as a feasible strategy to reduce the point-source CO_2_ emissions (Lackner, 2003; Reiner, 2016; Burant et al., 2017; Zhai et al., 2015; Sha et al., 2016; Bui et al., 2018; Wang, 2016). However, as the dispersed CO_2_ emissions account for 50% of total greenhouse emissions (Seipp et al., 2017), the application of point-source CCS technologies is unlikely to stabilize the atmospheric CO_2_ concentration at a desirable level (Sanz-Pérez et al., 2016). Accordingly, the concept of direct air capture (DAC) has been put forward which aims at capturing CO_2_ from ambient air (National Research Council, 2015; Keith, 2009; Sutherland, 2019; Jacobson, 2019; Bajamundia et al., 2019; Shi et al., 2020). In the past two decades, many efforts have been devoted for the development of various DAC sorbents, such as porous organic polymers (POPs), metal-organic frameworks (MOFs), solid-supported amine-based sorbents, and small molecular organic sorbents. Among them, the POPs are polymeric materials constructed by organic covalent bonds which are considered to be promising materials for CO_2_ storage due to the hyper-crosslinked structures and high stability (Zou et al., 2017; Li et al., 2017; Wang et al., 2017; Yang et al., 2019). The MOFs are highly porous materials constructed by metal cations and organic ligands which have large surface areas and can effectively trap carbon dioxide in the cages (Boyd et al., 2019; Trickett et al., 2017; González-Zamora and Ibarra, 2017). Solid-supported amine-based sorbents are solid adsorbents which incorporate amine moieties into solid supports including zeolites, carbons and organic resins, etc (Thakkar et al., 2017; Pang et al., 2017; Holewinski et al., 2017; Sarazen and Jones, 2017). These adsorbents offer advantages such as high selectivity for CO_2_ and relatively low cost, but they displayed lower CO_2_ capacities compared to other sorbents. In recent years, some specially designed organic compounds have shown great potential for DAC. For example, in 2014, Hossain group developed an organic compound with six urea groups which absorbed atmospheric CO_2_ as CO_3_^2−^ via 12 strong N—H∙∙∙O bonds under mild conditions (Pramanik et al., 2014). In 2017, Custelcean et al. reported an innovative iminoguanidine type sorbent, namely 2,6-pyridine-bis(iminoguanidine) (PyBIG) which could capture CO_2_ from ambient air, crystallize as an insoluble carbonate and regenerate PyBIG by mild heating with concomitant CO_2_ releasing (Seipp et al., 2017; Brethomé et al., 2018). Soon after, they disclosed another simple and robust iminoguanidine compound called glyoxal-bis(iminoguanidine) (GBIG), by which the flue gas CO_2_ absorption led to the formation of a dehydrated bicarbonate salt (Williams et al., 2019; Garrabrant et al., 2019); however, further results proved that the GBIG was ineffective for DAC (Custelcean et al., 2020). In addition, their most recent structure-property relationship study of GBIG, MGBIG (methylglyoxal-bis(iminoguanidine)) and DABIG (diacetyl-bis(iminoguanidine)) revealed that minor modifications in the molecular structures would result in dramatic differences in the crystal structures, aqueous solubilities, conformational flexibilities, as well as free energies for CO_2_ absorption (Custelcean et al., 2020). Although great progress has been made, the development of DAC sorbents for CO_2_ capture is still in its infancy. Thus, strategies and solutions for DAC processes that are facile, efficient, economical, independent of fossil resources, and applicable on a large scale would be highly desired.

From the viewpoints of green chemistry and climate change, the chemical conversion and utilization of biomass represent a kind of carbon neutral processes which would be more eco-friendly compare with the utilization of fossil resources (Chheda et al., 2007; Ji et al., 2018; Strube et al., 2011). As an indirect source of solar energy, biomass is abundant and renewable in nature. It is generally accepted that large-scale and efficient utilization of biomass would cause no net increase of carbon and would exert beneficial effects against global warming (Chheda et al., 2007; He et al., 2019; Ma et al., 2011).

**Figure 1:**
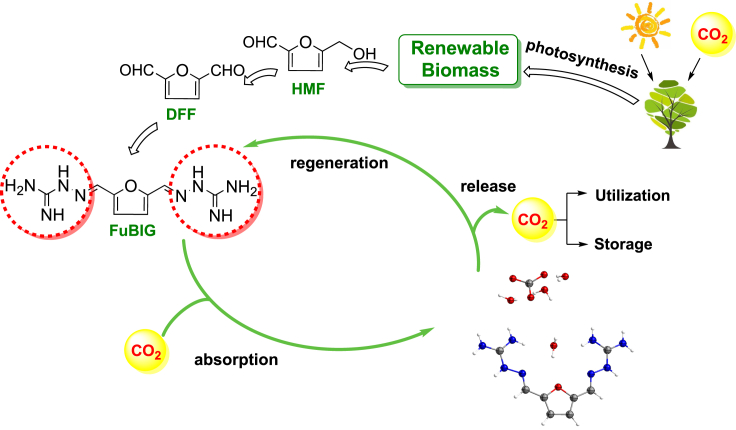
Illustration of the design, synthesis of the biomass-derived sorbent FuBIG showing negative emission properties following the concept of BBDAC

Platform molecules are recognized as vital hallmarks of biomass conversion and utilization which can be utilized as starting materials or building blocks for the production of a great number of downstream chemicals (Serrano-Ruiz et al., 2011; Arias et al., 2020; Luterbacher et al., 2014; Zhu et al., 2017). Among them, the 5-hydroxymethylfurfural (5-HMF) is one of the most versatile and highly transformable bio-based platform molecules originated from lignocellulose (Ma et al., 2011; Zhu et al., 2017; Saha and Omar, 2014; Solanki and Rode, 2019; Zhao et al., 2007). The partial oxidized derivative of 5-HMF, namely, 2,5-diformylfuran (DFF) has also been widely used as a starting material for the synthesis of pharmaceuticals, macrocyclic ligands, and functional polymeric materials (Ghatta et al., 2019; Zhang and Huber, 2018; Zhao et al., 2018). Inspired by the concept of bioenergy with carbon capture and storage (BECCS) (Davis et al., 2018), and as part of our continuing efforts on biomass conversion and utilization (Wu et al., 2012; Luo et al., 2019; Huang et al., 2018; Sheng et al., 2016; Zhang et al., 2014a, 2014b, 2014c, 2015), we envisioned that the concept of biomass conversion and DAC could be combined to the description of biomass-based direct air capture (BBDAC, Figure 1.). More specifically, we envisaged that the development of an iminoguanidine type of CO_2_ sorbents based on biomass-derived platform molecules would be rational and more favorable to achieve the goal of negative emissions. Herein, we report the design, synthesis, physicochemical study, and swing property of an efficient CO_2_ sorbent, the 2,5-furan-bis(iminoguanidine) (FuBIG), starting from the biomass-derived DFF.

## Results and discussion

### Synthesis, physical properties and crystallization analysis of FuBIG

**Figure 2:**
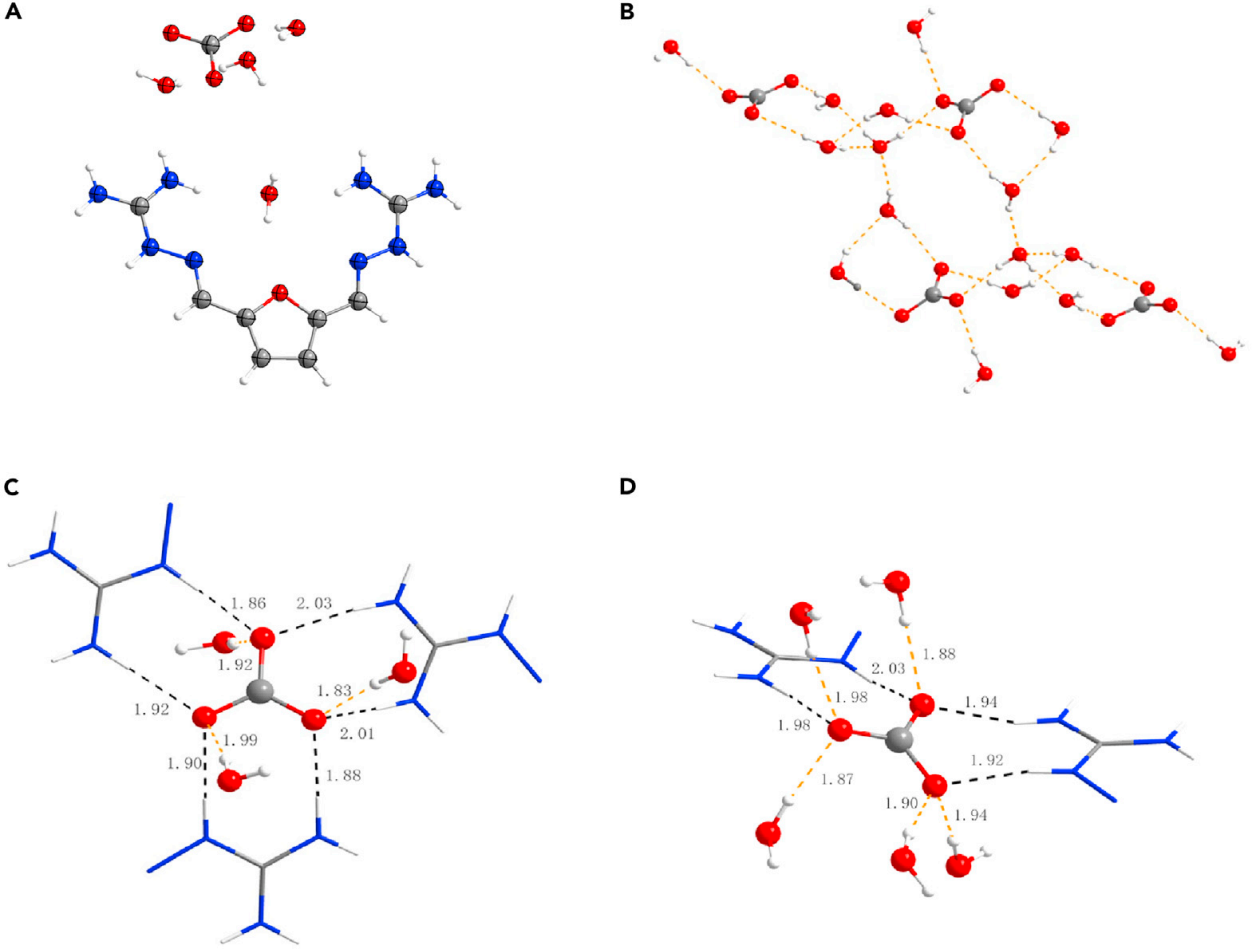
Atmospheric CO_2_ capture by FuBIG lead to the crystallization of FuBIGH_2_(CO_3_) (H_2_O)_4_ and the X-Ray crystal structure analysis (A) X-ray single crystal structure of FuBIGH_2_(CO_3_) (H_2_O)_4_ with 50% ellipsoids. (C, gray; H, white; N, blue; O, red.). (B) Ladder-shaped hydrogen-bonded [(CO_3_) (H_2_O)_4_]_n_ anionic cluster. (C) Binding mode A: each carbonate anion in the cluster accepted three water hydrogen bonds and six guanidinium hydrogen bonds. The FuBIGH_2_^2+^ cations have been truncated at the imine bond. (D) Binding mode (B): each carbonate anion in the cluster accepted five water hydrogen bonds and four guanidinium hydrogen bonds.

The FuBIG was readily obtained by the imine condensation of the biomass-derived DFF with aminoguanidinium chloride, followed by neutralization with aqueous NaOH. Gratifyingly, the FuBIG showed an improved aqueous solubility (0.4029 M, 25°C) than PyBIG (0.0012 M, 25°C), and when the aqueous solution of FuBIG was left open to ambient air for a few days, the formation of prism shaped, yellowish-brown single crystals was found, which was consistent with the composition of a tetrahydrated carbonate FuBIGH_2_(CO_3_) (H_2_O)_4_ by Fourier transform infrared spectroscopy (FTIR), elemental analysis (EA), and single-crystal X-ray diffraction analysis (CCDC: 2038310, Table S1, Figures S8 and 2A). Moreover, results showed that the ladder-shaped [(CO_3_^2−^) (H_2_O)_4_]_n_ anionic cluster was formed and extended through hydrogen-bonds (Figure 2B). Interestingly, it was found that there were a total of nine hydrogen bonds with two types of binding modes (A and B) existing in every two adjacent carbonate anion in the crystal structures of FuBIGH_2_(CO_3_)(H_2_O)_4_. In binding mode A (Figure 2C), each carbonate anion in the cluster accepted three water hydrogen bonds to form [CO_3_(H_2_O)_4_^2-^]_n_ clusters, with O—H∙∙∙O contact distances ranging between 1.83 and 1.99 Å, and with bond angles between 170° and 172°. Furthermore, each carbonate anion also accepted another six guanidinium hydrogen bonds, with N—H∙∙∙O contact distances ranging between 1.88 and 2.03 Å, and bond angles between 157° and 176°. In binding mode B (Figure 2D), the carbonate anion accepted five hydrogen bonds from water with O—H∙∙∙O contact distances ranging between 1.87 and 1.98 Å, and with bond angles between 166° and 177°. Correspondingly, the carbonate anion accepted another four guanidinium hydrogen bonds, with N—H∙∙∙O contact distances ranging between 1.92 and 2.03 Å, and with bond angles between 157° and 168°. Single-crystal X-ray diffraction analysis of FuBIGH_2_(CO_3_)(H_2_O)_4_ displayed that the hydrogen bonding between FuBIG, CO_2_ (as CO_3_^2-^), and H_2_O was significant and would be the main driving force for the capture of CO_2_ in ambient air. Moreover, the energy of the hydrogen bonds has been analyzed by DFT study. The energy of the hydrogen bonds between FuBIGH_2_^2+^ and CO_3_^2-^ is calculated to be 16.4 kcal/mol, and the energy of the hydrogen bonds between FuBIGH_2_(CO_3_) and H_2_O is calculated to be 6.5 kcal/mol (see Table S12: The cartesian coordinates (xyz) for hydrogen bonds on DFT calculation for details). These results suggest that hydrogen bonds would provide a delicate balance between the stability of FuBIGH_2_(CO_3_)(H_2_O)_4_ and the property of FuBIG regeneration and CO_2_ release.

### Thermodynamic and kinetic analysis of CO_2_ absorption and heat release

**Scheme 1:**
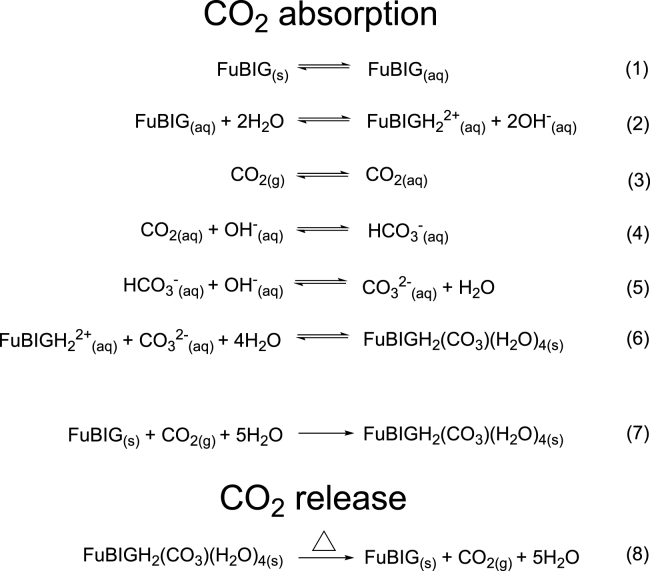
Stepwise and overall reactions in CO_2_ capture process of FuBIG and CO_2_ heat release of its carbonate salt

The thermodynamic and kinetic study of CO_2_ absorption and heat release are crucial tasks in the search for CO_2_ sorbents, which could provide accurate physicochemical parameters and minimum energy requirements for the reactions in stepwise and overall manner, thereby paving the way for further optimization and application. The reactions involved in the CO_2_ absorption and heat release regarding FuBIG are shown in Scheme 1, and the corresponding thermodynamic parameters are listed in Table 1.

**Table 1:** Thermodynamic and kinetic parameters for CO_2_ capture and heat release

Entry	Reaction/parameter	ΔH (kJ/mol)^c^	K^c^	Solubility (mol/L)^c^	Reference
1	FuBIG dissolution	ΔH_1_ = 49.19^d^	K_1_ = 4.03 × 10^−1^^i^	S_1_ = 0.4029	Figure S10
2	FuBIG protonation^a^	ΔH_2_ = 46.08^e^	K_2_ = 1.91 × 10^−12^^j^	–	Figure S12
3	CO_2_ dissolution	ΔH_3_ = −19.4	K_3_ = 3.4 × 10^−2^	–	Carroll et al., 1991
4	HCO_3_^-^ formation	ΔH_4_ = −50	K_4_ = 3.02×10^7^	–	Wang et al., 2010
5	CO_3_^2−^ formation	ΔH_5_ = −40.4	K_5_ = 4.66×10^3^	–	McCann et al., 2011
6	FuBIGH_2_(CO_3_) (H_2_O)_4_ crystallization	ΔH_6_ = −101.57^f^	K_6_ = 1.63×10^7^^k^	S_6_ = 0.009344	Figure S11
7	overall CO_2_ absorption	ΔH_7_ = −116.1^g^	K_7_ = 5.97 × 10^4^^l^	–	Table 1
8	CO_2_ release^b^	ΔH_8_ = 209.31^h^	–	–	Figure S15
9	[R_s_]	–	–	43.12^m^	Figures S10 and S11

a This reaction included a double deprotonation processes of water.

b CO_2_ heat release was measured at a persistent heating rate (10°C/min).

c The parameters were determined at standard temperature (25°C) unless otherwise specified.

d Determined by van't Hoff analysis of solubility values of FuBIG measured in the 15–35°C range.

e The enthalpies for the protonation processes of FuBIG were calculated by van't Hoff analysis of pK_a_ values measured in the 15–35°C range, and included the enthalpies for a double deprotonation processes of water (55.8 kJ/mol).

f Determined by van't Hoff analysis of K_sp_ values measured in the 15–35°C range.

g ΔH_7_ = ΔH_1_ + ΔH_2_ + ΔH_3_ + ΔH_4_ + ΔH_5_ + ΔH_6_.

h Determined from the endotherm observed in the DSC.

i K_1_ = K_sp_(FuBIG).

j K_2_ = (K_w_)^2^/(K_a1_ × K_a2_).

k K_6_ = 1/K_sp_(FuBIGH_2_(CO_3_) (H_2_O)_4_).

l K_7_ = K_1_×K_2_×K_3_×K_4_×K_5_×K_6_.

m [R_s_] = S_1_/S_6_.

The reactions involved in the CO_2_ absorption comprise six steps represented by Equations (1)–(6) in Scheme 1. First, FuBIG dissolves into water (Equation 1), followed by acceptance of two protons from water to generate the FuBIGH_2_^2+^ cations and OH^−^ (Equation 2). Then, CO_2_ transports from air into aqueous solution (Equation 3) and reacts with OH^−^ to generate HCO_3_^-^ (Equation 4) and subsequently give CO_3_^2−^ (Equation 5). Finally, the FuBIGH_2_^2+^ and CO_3_^2−^ ions crystallize with water to afford FuBIGH_2_(CO_3_) (H_2_O)_4_ (Equation 6).

The corresponding enthalpy (ΔH) values for Equations (1)–(6) are listed in Table 1, the enthalpies of CO_2_ dissolution (Equation 3, ΔH_3_ = −19.4 kJ/mol), and the generation of HCO_3_^-^ and CO_3_^2−^ (Equations 4 and 5, ΔH_4_ = −50 kJ/mol, ΔH_5_ = −40.4 kJ/mol) were obtained from previous studies (Carroll et al., 1991; Wang et al., 2010; McCann et al., 2011). The remaining enthalpies for the reactions including FuBIG dissolution (Equation 1) and FuBIGH_2_(CO_3_) (H_2_O)_4_ crystallization (Equation 6) were determined by van't Hoff analysis of variable temperature solubility assay, respectively (ΔH_1_ = −49.19 kJ/mol, ΔH_6_ = −101.57 kJ/mol, Figures S11 and S12, Tables S4 and S5). Similarly, the enthalpy for FuBIG protonation (Equation 2) were obtained from van't Hoff analysis of variable temperature pK_a_ assay (ΔH_2_ = −46.08 kJ/mol Table S7 and Figure S13). The overall reaction of CO_2_ absorption is represented by Equation 7 (Scheme 1), which has an overall enthalpy (ΔH_7_) of −116.10 kJ/mol. These results showed that the overall enthalpy value was more negative from FuBIG to FuBIGH_2_(CO_3_) (H_2_O)_4_ than that from PyBIG to PyBIGH_2_(CO_3_) (H_2_O)_4_ (−70.7 kJ/mol) (Brethomé et al., 2018), indicating that the CO_2_ absorption of FuBIG was more exothermic and would be easier to take place than that of PyBIG.

The overall equilibrium constant for CO_2_ absorption is 5.97×10^4^ (K_7_ = K_1_×K_2_×K_3_×K_4_×K_5_×K_6_, Table 1). It is worth-mentioning that the values of K_3_, K_4_ and K_5_ are invariable under ideal conditions, so the extent of the overall reaction of any specially designed aminoguanidine sorbent could be improved with the increase of basicity (K_2_) and solubility of the free base (K_1_) and the decrease of solubility of its carbonate salt (K_6_). Specifically, strong basicity of the sorbent would facilitate the transformation of CO_2_ to CO_3_^2−^, high solubility of the free base would make the aqueous solution of the sorbent to interact with the gaseous CO_2_ more efficiently, whereas the low solubility of the corresponding carbonate salt would lead to the precipitation more thorough and the separation of which by filtration more convenient. However, as the differences of basicity between iminoguanidine sorbents are generally indistinctive, therefore, the solubility of the free base and its carbonate salt would be the simple and determining factors for this kind of sorbents. Accordingly, The value [R_s_] was introduced in this study which was defined as the solubility ratio between FuBIG and its carbonate FuBIGH_2_(CO_3_) (H_2_O)_4_ at 25°C ([R_s_] = 43.12, Table 1, Tables S4 and S5). Moreover, according to solubility data reported by Custelcean et al. (Brethomé et al., 2018; Williams et al., 2019), the [R_s_] value for PyBIG and GBIG were determined to be 7.97 and 1.60, respectively. Thus, the results suggested that the FuBIG would be more favorable as a CO_2_ sorbent than PyBIG and GBIG in terms of [R_s_] value.

**Figure 3:**
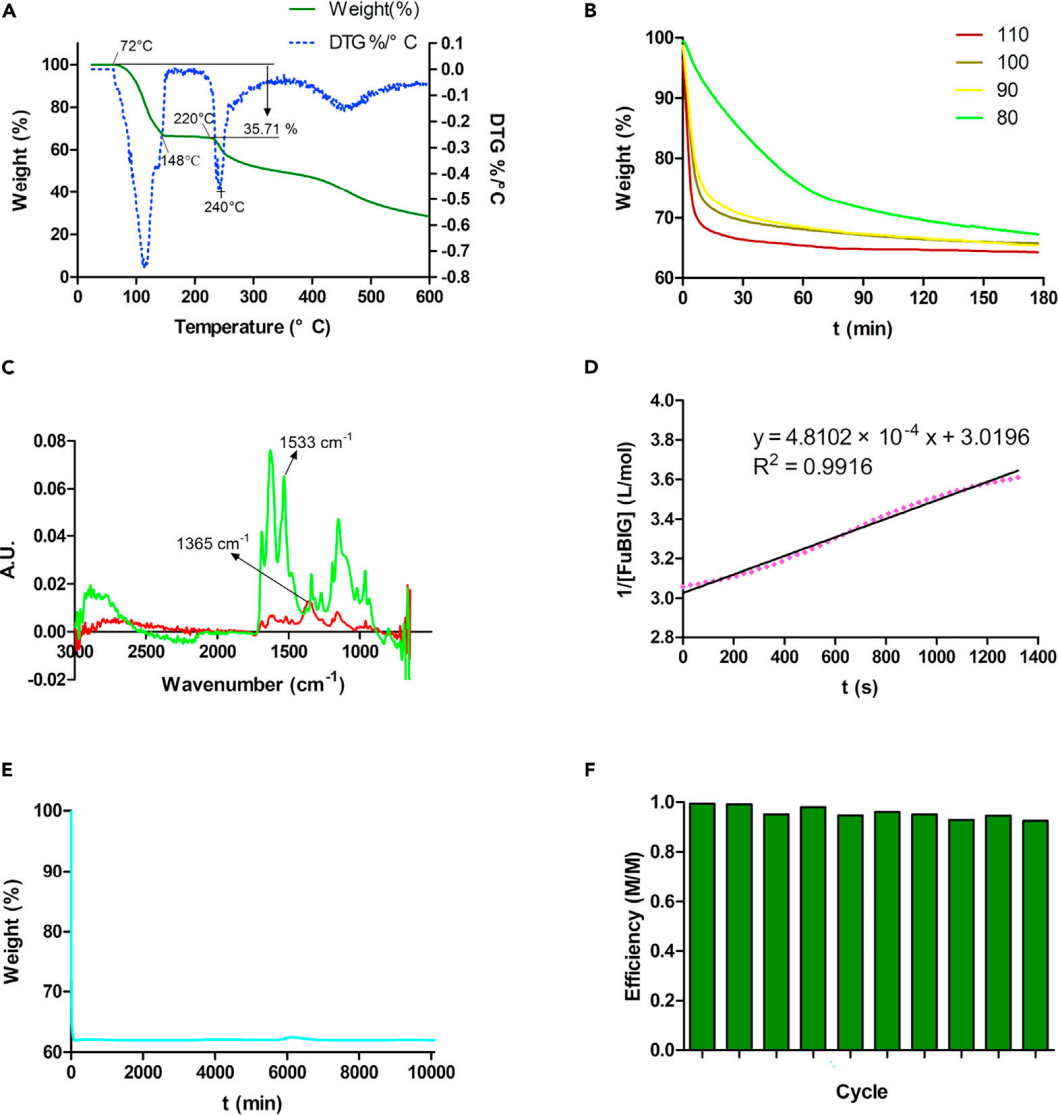
Thermodynamic and kinetic analysis of CO_2_ absorption and release with FuBIG and applicability evaluation in practical process (A) Temperature-ramped TGA plots showing CO_2_ and H_2_O release from FuBIGH_2_(CO_3_) (H_2_O)_4_. (B) Isothermal TGA data used for the kinetic analysis of CO_2_ release from FuBIGH_2_(CO_3_) (H_2_O)_4_ at 80°C, 90°C, 100°C, 110°C. (C) Infrared absorption of FuBIG (green) and its carbonate (red), the peak at 1533 cm^−1^ is the characteristic N-H absorption of FuBIG, while the peak at 1365 cm^−1^ representing the wavenumber of the carbonate salt. (D) The simulated pattern of reaction order for CO_2_ absorption with FuBIG at 25°C. (E) Isothermal (110°C) thermogravimetric analysis of FuBIGH_2_(CO_3_) (H_2_O)_4_ in air over a one-week period. (F) Efficiency of CO_2_ sorbent (M/M) over ten consecutive absorption-release cycles.

When the crystals of FuBIGH_2_(CO_3_) (H_2_O)_4_ were heated in an oven at 120°C for 1h, the crystals transformed to FuBIG and changed their appearance from transparent to opaque, while maintaining the original yellow color (Figure S15). Thermogravimetric analysis (TGA) provided a quantitative measurement of the decomposition process (Figure 3A). FuBIGH_2_(CO_3_) (H_2_O)_4_ showed a 35.71% mass loss between 72°C and 148°C, which was consistent with the loss of 1 equiv. of carbonic acid (as CO_2_ and H_2_O) and 4 equiv. of H_2_O (36.22% theoretical mass loss). After the complete regeneration of FuBIG at 148°C, the TGA curve became flat with the increase of temperature to about 220°C, this result indicated that there was no thermal decomposition occurred in this temperature range, demonstrating a high thermostability of FuBIG. Subsequently, it was found that the FuBIG began to decompose at around 240°C with further increment of temperature. In addition, the very similar TGA pattern between FuBIGH_2_(CO_3_) (H_2_O)_4_ and FuBIG at above 140°C, fully confirmed the regeneration process from FuBIGH_2_(CO_3_) (H_2_O)_4_ to FuBIG under thermal conditions (Figures 3A and S14). Overall, the CO_2_ capture of the biomass-based FuBIG from ambient air and heat release of CO_2_ with concomitant regeneration of the sorbent provided direct experimental evidence for the feasibility of BBDAC.

The differential scanning calorimetry (DSC) analysis of FuBIGH_2_(CO_3_) (H_2_O)_4_ displayed an endothermic pattern corresponding to the loss of CO_2_ and H_2_O in TGA study, with a measured releasing enthalpy (ΔH_8_) of 209.31 kJ/mol (Figure S16). Before the temperature reached the point for CO_2_ release, it could be calculated from the DSC measurements that an enthalpy of 48.40 kJ/mol was needed for the specific heat capacity of FuBIGH_2_(CO_3_) (H_2_O)_4_ (Figure S17). Hence, the total enthalpy requirement for the regeneration of FuBIG would be 257.71 kJ/mol.

Isothermal TGA running at constant temperatures of 40, 50, 60, 70, 80, 90, 100, and 110°C respectively, provide evidence for the kinetic analysis of the CO_2_ and H_2_O released from FuBIGH_2_(CO_3_) (H_2_O)_4_ (Figure S18). Among them, it was found that the practical and effective data was obtained from temperature range of 80, 90, 100, and 110°C (Figure 3B). After plotting the fractional conversion (α) as a function of time (Figure S19), the most common solid-state reaction kinetics, including Avrami-Erofeev, Prout-Tompkins, Ginstling-Brounstein, Jander, and geometrical phase-boundary models were screened (Figure S20). It was found that the most fitted model would be assigned to the geometrical phase-boundary model characterized by Equation (9):

(Equation 9) $$1-{\left(1-\alpha \right)}^{1/3}=kt,\;\alpha =\left(m_{0}-m\right)/\left(m_{0}-m_{f}\right)$$

Where m_0_ represents the initial sample weight of FuBIG carbonate salt, m_f_ represents the final weight after isothermal heating, and m represents the sample weight at a certain time (Figure S20). This result revealed that the reaction initiated on the surface of crystals, followed by inward advance to the center, and resulting in a decelerator α-t curve with the decrease of interface. Moreover, activation barrier (E_a_ = 92.24 kJ/mol) for the decomposition of FuBIGH_2_(CO_3_) (H_2_O)_4_ was obtained from Arrhenius analysis of the rate constants (k) under different temperatures (Figure S21).

The recycling kinetics of CO_2_ absorption and release corresponding to the transformation of FuBIG—FuBIGH_2_(CO_3_) (H_2_O)_4_—FuBIG could be characterized by *in situ* React IR in solution state. The characteristic wavelength of FuBIG and FuBIGH_2_(CO_3_) (H_2_O)_4_ were found to be 1533 cm^−1^ (N—H) and 1365 cm^−1^ (CO_3_^2−^), respectively (Figure 3C). By monitoring the intensity change of these absorption peaks, the corresponding concentration changes of FuBIG and FuBIG carbonate salt could be obtained. As shown in Figure S23, when CO_2_ was put into the aqueous solution of FuBIG, the concentration of FuBIG decreased with the formation of FuBIG carbonate salt (the concentration change of carbonate anion was relatively complicated owing to the multifactorial influence toward the labile FuBIG carbonate salt under thermal conditions in solution state). By analyzing the concentration-time curve of FuBIG with integral method, it could be found that the process of CO_2_ absorption was in accordance with the second order reaction kinetics with a rate constant (k) of 4.8102 × 10^−4^ L/mol·s at 25°C (Figure 3D).

### Applicability evaluation of FuBIG in practical process

It is widely accepted that the recycling of both CO_2_ and the sorbent is indispensable in practical CCUS process (Gao et al., 2020; Flores-Granobles and Saeys, 2020; Leclaire and Heldebrant, 2018; Gonzalez-Diaz et al., 2020). Therefore, in this study, the crystals of FuBIGH_2_(CO_3_) (H_2_O)_4_ were heated at 110°C in oven for one week for the investigation of the robustness of FuBIG in CO_2_ capturing and releasing process. The weight was measured every 6 hr. After the release of CO_2_ and H_2_O, FuBIG showed no sign of decomposition (Figure 3E). The weight of the solid was fluctuating within a narrow range.

Next, we ran a full CO_2_ separation cycle using CO_2_ balloon to assess the recyclability of this sorbent. CO_2_ gas was injected into the saturated aqueous solution of FuBIG, leading to the formation of yellow precipitate within minutes. The solid was collected by filtration and the filtrate was analyzed by ultraviolet-visible spectroscopy to determine the concentration of the free FuBIG left in the solution. At the first cycle, 99.36% of FuBIG was converted to the FuBIG carbonate salt. Then the FuBIG carbonate salt was heated at 110°C for 4h, leading to complete release of CO_2_ and H_2_O. The regenerative FuBIG was then dissolved into the filtrate and precipitated again by CO_2_. Overall, ten consecutive CO_2_ capture/release cycles were conducted, and the conversion rate could still maintain at 92.49% (Figure 3F). Although the long-term stability and recyclability of FuBIG remains to be explored over more and more capture/release cycles under practical conditions, our preliminary results indicated that this biomass-derived sorbent is remarkably robust.

The transformation of CO_2_ into bulk chemicals or value-added products represents an attractive strategy for CO_2_ utilization. In our previous work, a facile and operationally simple method (room temperature, 1 atm of CO_2_ balloon) for the synthesis of various *O-*aryl carbamates via one-pot three-component coupling of aryl carboxamides, CO_2_, and amines has been established (Luo et al., 2019). We reasoned that the CO_2_ captured by biomass-derived sorbent FuBIG could be utilized in chemical reactions. Accordingly, in this study, a round bottom flask filled with FuBIGH_2_(CO_3_) (H_2_O)_4_ in place of CO_2_ balloon was connected with the reaction system, the CO_2_ airflow was stably generated at 80°C, and reacted with aryl carboxamide and amine to afford the desired *O-*aryl carbamate in 70% yield in the presence of CuI and MnO_2_ (Figure S24) (Luo et al., 2019). This result demonstrated the feasibility for the circulation of CO_2_ capture, release and utilization regarding the transformation between FuBIG and FuBIG carbonate salt and represented a practical example for negative emissions under the proposed BBDAC rationale.

### Spontaneous CO_2_ release of FuBIG carbonate salt in DMSO

**Figure 4:**
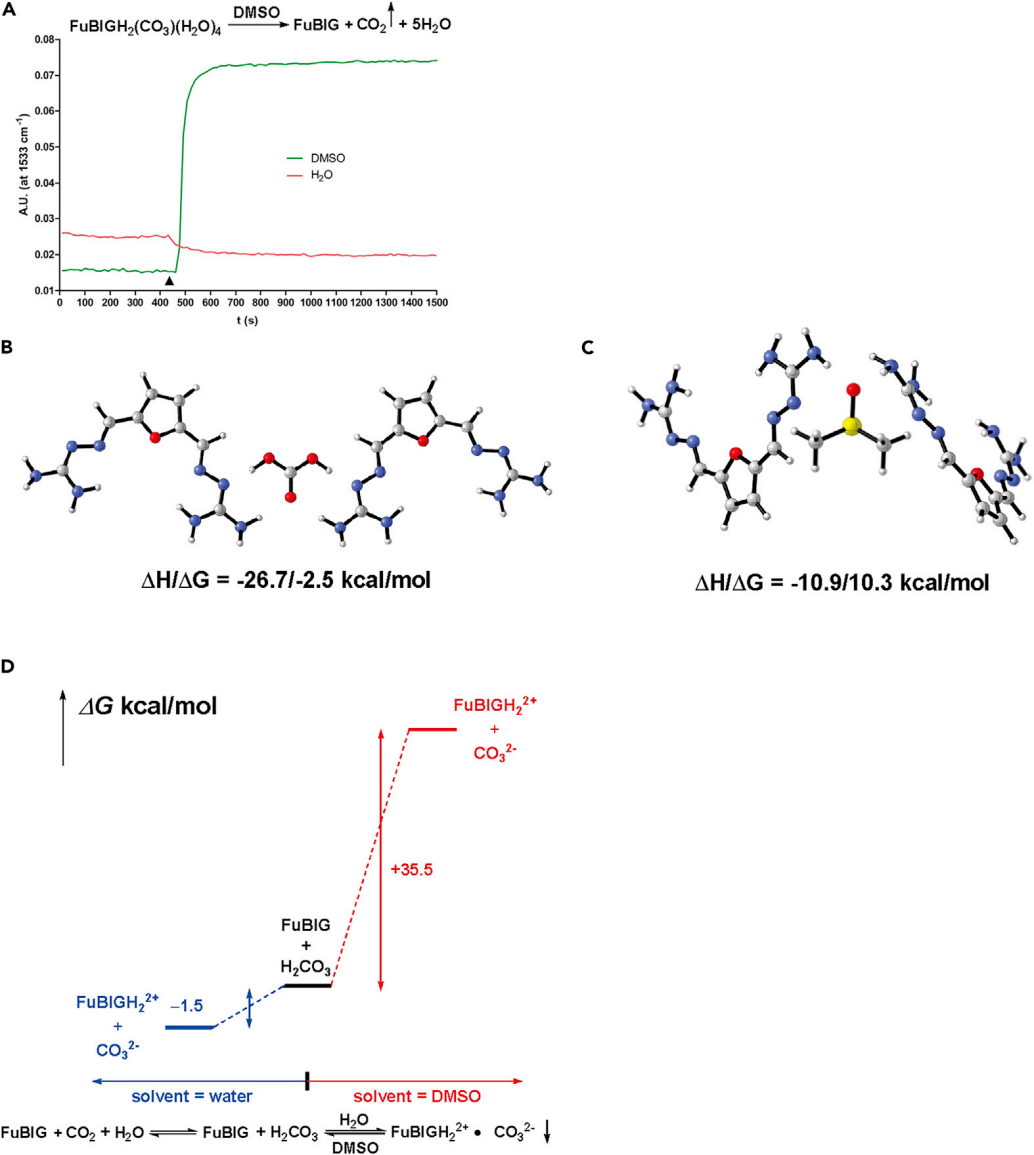
Spontaneous CO_2_ release of FuBIG carbonate salt in DMSO (A) Absorption intensity monitored at 1533 cm^−1^ (N—H) in liquid phase using React IR when FuBIG carbonate salt being added into DMSO and H_2_O respectively. Triangle: representing the time point for the adding of FuBIG carbonate salt into the solvent. (B) Binding energy of 2eq FuBIG with H_2_CO_3_. (C) Binding energy of 2eq FuBIG with DMSO. (D) Potential energy surface of the forming of FuBIGH_2_^2+^ and CO_3_^2-^.

The regeneration convenience and long-term robustness of CO_2_ sorbents are vital factors in the development of practical and efficient DAC technologies. It is quite obvious that, for an optimal CO_2_ sorbent, large energy requirement should be avoided for CO_2_ release and sorbent regeneration. However, to the best of our knowledge, current DAC technologies including humidity swing, pressure swing, or temperature swing, are energy-intensive, strongly endothermic and complicated in the operating processes. Surprisingly and delightfully, during the recording of the NMR spectra for FuBIG carbonate salt, some interesting results displaying a unique, unprecedented, and spontaneous way for CO_2_ release from FuBIG carbonate salt were obtained. It was found that when DMSO-d_6_ was added into the FuBIG carbonate salt with oscillation at room temperature, the insoluble solid gradually dissolved with concomitant release of CO_2_ in an endothermic manner in a few minutes. The ^1^HNMR and ^13^CNMR spectra gave identical results compared with that of FuBIG, demonstrating a unique and easy way for CO_2_ release and regeneration of FuBIG from its carbonate salt in DMSO-d_6_ (Figures S4 and S5). Subsequently, the non-deuterated DMSO was tested (showed by React IR in Figure 4A), and the same result was obtained. Amazingly, other commonly used solvents such as tetrahydrofuran, ethanol, methanol, ethyl acetate, and chloroform etc. were unable to regenerate FuBIG from its carbonate salt under the same conditions. Our findings regarding the spontaneous CO_2_ release of FuBIG carbonate salt in DMSO/DMSO-d_6_ might represent a technique with near-zero energy input and might open up a new avenue for DAC technologies.

To gain further insight into the spontaneous process for CO_2_ release of FuBIG carbonate salt in DMSO, calculation studies were carried out by density functional theory (Hohenberg and Kohn, 1964; Kohn and Sham, 1965). As illustrated in Figures 4B and 4C, owing to the existence of hydrogen bonds, the binding energy representing by ΔH and ΔG between H_2_CO_3_ and FuBIG are more negative in value than that of between DMSO and FuBIG (for interaction of H_2_CO_3_ with FuBIG: ΔH/ΔG = −26.7/-2.5 kcal/mol; for interaction of DMSO with FuBIG: ΔH/ΔG = −10.9/10.3 kcal/mol). Therefore, the extrusion of CO_2_ from FuBIG carbonate salt in DMSO should not result from stronger interaction between DMSO and FuBIG. Moreover, as shown in Figure 4D, the reaction between H_2_CO_3_ and FuBIG in water to form FuBIG carbonate salt is spontaneous (ΔG = −1.5 kcal/mol), whereas the same reaction taken place in DMSO is nonspontaneous (ΔG = 35.5 kcal/mol) (see Table S13: The cartesian coordinates (xyz) for all optimized structures on DFT calculation for details). Accordingly, it could be concluded that H_2_CO_3_ and FuBIG are more in inclined to form the ion pair (FuBIGH_2_^2+^⋅CO_3_^2−^) in water, while the counterreaction of which is more likely to take place in DMSO.

Interestingly, we have also found that the DMSO solution saturated with FuBIG could still promote the release of CO_2_ from FuBIG carbonate, and result in the regeneration of FuBIG. This intriguing result indicated that a dynamic equilibrium might be achieved for CO_2_ release and FuBIG regeneration in these conditions, thereby providing a proof of concept for a highly efficient and energy-saving protocol for the cycling of CO_2_ capture, release and sorbent regeneration. It is obvious that under these conditions, the recovery of FuBIG (by filtration) from DMSO could be realized spontaneously and continuously with minimum DMSO consumption, and evaporation of DMSO is no longer needed for the sorbent recovery (Figures S25 and S26). In addition, freeze drying might be another option for DMSO removing and sorbent recovery.

### Biocompatibility assay of FuBIG

**Figure 5:**
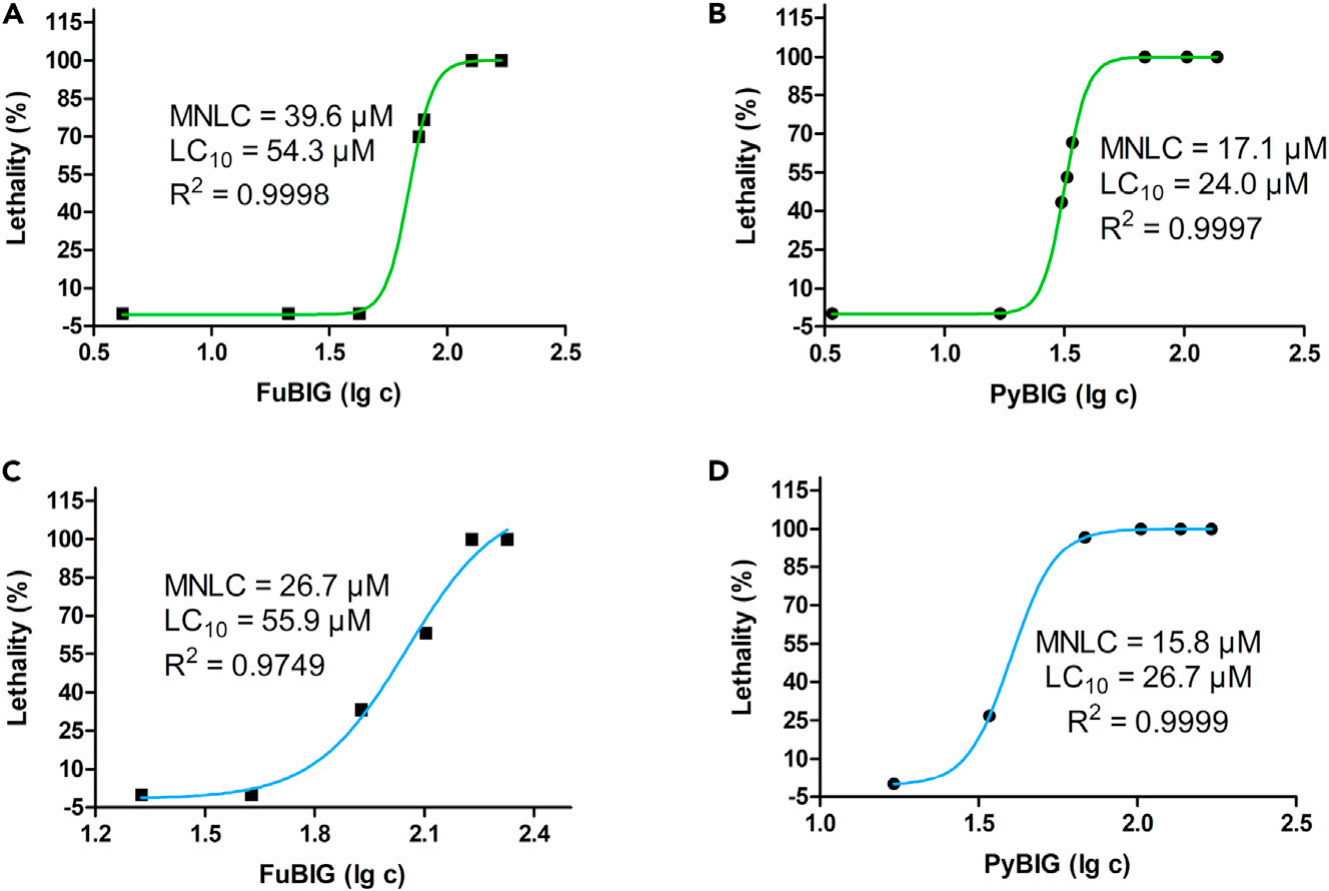
Biocompatibility assay of FuBIG and PyBIG in zebrafish (A) Acute toxicity test of FuBIG with lethality (%) versus log function of concentration (μM) plots. (B) Acute toxicity test of PyBIG with lethality (%) versus log function of concentration (μM) plots. (C) Embryo toxicity test of FuBIG with lethality (%) versus log function of concentration (μM) plots. (D) Embryo toxicity test of PyBIG with lethality (%) versus log function of concentration (μM) plots.

The future application scenarios of DAC sorbents could be classified into two categories: exceptionally large-scale deployments for CO_2_ capture either from point-sources or from ambient atmosphere, and small-scale facilities for CO_2_ capture in enclosed cabins such as space capsules or submarines. Accordingly, the biocompatibility properties of DAC sorbents should be taken into consideration. In this study, zebrafish were used as the model species, the tests of acute toxicity and embryo toxicity were conducted, respectively, for the biocompatibility evaluation of FuBIG and the previously reported PyBIG. Results showed that the FuBIG was less toxic than PyBIG in acute toxicity test in terms of maximum non-lethal concentration (MNLC) and 10% lethal concentration (LC_10_), with the corresponding measurements for FuBIG (MNLC = 39.6 μM, LC_10_ = 54 μM) and PyBIG (MNLC = 17.1 μM, LC_10_ = 24 μM), respectively (Figures 5A and 5B). In target organ toxicity tests of FuBIG (1.9, 5.7, 17.1, and 24.0 μM), no toxicity was observed in 1.9 and 5.7 μM groups, whereas the delay of yolk sac absorption was found in 16.7% and 23.3% of zebrafish in 17.1 μM and 24.0 μM groups, respectively. No other toxicity was found in all of the FuBIG groups. By comparison, for zebrafish groups treated with PyBIG (1.9, 5.7, 17.1, and 24.0 μM), no toxicity was observed in 1.9 μM group, whereas the delay of yolk sac absorption was found in 13.3% of zebrafish in 5.7 μM group. Moreover, it was observed that in 17.1 μM group treated with PyBIG, 6.7–30.0% of zebrafish developed renal edema, pericardium edema, delay of yolk sac absorption, lack and slowing down of blood flow, respectively. Further increment of PyBIG concentration to 24.0 μM, more serious toxic responses was observed with 10.0% of deaths occurred. In addition, FuBIG was found to be less toxic than PyBIG in embryo toxicity assay (for FuBIG: MNLC = 26.7 μM, LC_10_ = 55.9 μM; for PyBIG: MNLC = 15.8 μM, LC_10_ = 26.7 μM. Figures 5C and 5D). Notably, target organ toxicity tests of FuBIG and PyBIG in the same concentrations (1.8, 5.3, 15.8, and 26.7 μM) also revealed that the FuBIG has less embryo toxicity than PyBIG. The above-mentioned results suggested that the biomass-derived CO_2_ sorbent might be more eco-friendly and more favorable in biocompatibility.

### Conclusion

In summary, based on the concept of BBDAC, the biomass-based facile synthesis and comprehensive evaluation of the CO_2_ chemical sorbent FuBIG are reported. Two binding modes with nine hydrogen bonds between FuBIG, CO_2_ (as CO_3_^2−^), and H_2_O were determined by single-crystal X-ray diffraction analysis. The stepwise and overall thermodynamic and kinetic parameters for CO_2_ absorption and heat release have been obtained through van't Hoff analysis, TGA, DSC and *in situ* reaction analysis. The reaction for CO_2_ absorption has an overall enthalpy value (ΔH_7_) of −116.10 kJ/mol, and an overall equilibrium constant (K_7_) of 5.97×10^4^, showing that the absorption of CO_2_ in aqueous solution of FuBIG is highly advantageous. Moreover, the reaction for CO_2_ heat release of FuBIGH_2_(CO_3_) (H_2_O)_4_ displayed a relatively lower energy requirement with an enthalpy value (ΔH_8_) of 209.31 kJ/mol. Besides, a simple and intuitive symbol for the evaluation of CO_2_ sorbents, namely, the [R_s_] value which was defined as the solubility ratio between sorbents and their carbonate salts was proposed in this study ([R_s_] = 43.12 for FuBIG, 7.97 for PyBIG, and 1.60 for GBIG, respectively, at 25°C). In addition, React IR analysis showed that the CO_2_ absorption process was consistent with the second-order reaction kinetics with a rate constant (k) of 4.8102 × 10^−4^ L/mol·s at 25°C, whereas the isothermal TGA analysis demonstrated that the kinetic characters for the release of CO_2_ and H_2_O from FuBIG carbonate salt was in line with the geometrical phase-boundary model. Notably, it was found that the spontaneous CO_2_ release of FuBIG carbonate salt occurred in DMSO, which might represent a near-zero-energy technique for DAC, this amazing process for spontaneous CO_2_ release in DMSO was further elucidated by DFT calculations. Finally, the acute toxicity and embryo toxicity assay in zebrafish model displayed that the biomass-derived CO_2_ sorbent of FuBIG was more favorable in terms of biocompatibility. Further investigations to develop more efficient biomass-derived sorbents and researches on structure-property relationships are underway in our group.

### Limitations of the study

This study designed and synthesized an iminoguanidine type CO_2_ sorbent, the FuBIG, starting from the biomass-derived platform compound DFF as the core structure. However, the side chain of iminoguanidine (transformed from aminoguanidine) was not biomass-sourced at present. Technically speaking, the aminoguanidine could be biomass-sourced when it is needed (see Figure S1 for details). In addition, the capacity of FuBIG decreases by 7% within 10 cycles, showing an insufficient efficiency in CO_2_ capture/release and sorbent regeneration process. Considering that the weight loss in sample transfer procedure was inevitable, especially at a laboratory scale, it is possible that when the experiment is performed at a large-scale, the weight loss in sample transfer process would be greatly reduced. Furthermore, techno-economic analysis and life cycle analysis are warranted for future work.

### Resource availability

#### Lead contact

Further information and requests for resources should be directed to and will be fulfilled by the lead contact, Yong Zou (zouyong3@mail.sysu.edu.cn).

#### Materials availability

Full experimental procedures are provided in the Supplemental Information.

#### Data and code availability

The accession numbers for FuBIGH_2_(CO_3_) (H_2_O)_4_ is CCDC: 2038310.

## Methods

All methods can be found in the accompanying transparent methods supplemental file.

## Supplemental information

Document S1. Transaprent methods, Figures S1–S28, and Tables S1–S11

Table S12. The cartesian coordinates (xyz) for hydrogen bonds on DFT calculation, related to Firure 2

Table S13. The cartesian coordinates (xyz) for all optimized structures on DFT calculation, related to Figure 4
