# Supplementary material for: Design, synthesis, and physicochemical study of a biomass-derived CO2 sorbent 2,5-furan-bis(iminoguanidine)
Source: iScience. 2021 Mar 4;24(4):102263. doi: 10.1016/j.isci.2021.102263 (PMC7995611; doi:10.1016/j.isci.2021.102263)
Supplement: Document S1. Transaprent methods, Figures S1–S28, and Tables S1–S11 [file mmc1.pdf]

iScience, Volume *24*

Supplemental information

Design, synthesis, and physicochemical study of a biomass-derived CO_2 sorbent 2,5-furan-bis(iminoguanidine)

Qianzhong Zhang, Yi Jiang, Yinwu Li, Xianheng Song, Xiang Luo, Zhuofeng Ke, and Yong Zou

Supplemental Information

Transparent Methods

Experimental

Common reagents used in the synthesis were obtained commercially and used without further purification unless otherwise specified. All water used was deionized (18 mΩ). The ^{1}H NMR and ^{13}C NMR spectra were recorded using TMS as the internal standard on a Bruker BioSpin GmbH spectrometer at 400, 500 MHz respectively. UV-vis spectra were measured in 10-mm-path-length quartz cuvettes using a UV-2600 (SHIMADZU). pH measurements were conducted with a PHS-3E pH meter (Shanghai INESA Scientific Instrument CO., ltd) using an E-301F pH electrode. PXRD measurements were performed with X-ray single crystal diffractometer (Xcalibur Nova). 12423 diffraction points and 5860 independent diffraction points (R_{int} = 0.0204, R_{sigma} = 0.0213) were collected within the range of $2\theta_{max}$ = 134° at T = 100 K using Cu-Kα ray (λ = 1.54184 Å) was used as a light source. The crystal structure was analyzed by XS (Sheldrick, 2008) and refined by SHELXL (Sheldrick, 2015). TGA was under a nitrogen atmosphere using a STA 409PC (NETZSCH). DSC measurements were conducted under nitrogen with a DSC 3 (METTLER TOLEDO). In situ reaction analysis was conducted with a React IR 15 (METTLER TOLEDO). Acute toxicity and embryo toxicity experiment in zebrafish were observed with stereomicroscope (SZX7, OLYMPUS, Japan), and photos taken by CCD camera (VertA1, Shanghai Tusen Vivion Technology Co., Ltd, China). Sorbents were weighed with a precision electronic balance (CP214, OHAUS, USA).

Methods

Synthesis of FuBIG

2,5-furandialdehyde (12.4 g, 0.1 mol), aminoguanidine hydrochloride (22 g, 0.2 mol) and ethanol (100 mL) were added to a 250 mL round bottom flask fitted with a condenser. The mixture was heated at 70 °C with stirring for 8 h. After the reaction was ended, the reaction mixture was subjected to standing for 4h at 4 °C. The $FuBIGH_2Cl_2$ product was collected by vacuum filtration as a light yellow solid, washed with ethanol for three times, then was dried under vacuum. The procedure yielded 34.7 g (98% yield). The chloride salt recrystallized from ethanol to be used in pK_a experiment.

The above 2,5-furyldiiminoguanidine hydrochloride hydrate was put in a 250 mL round bottom flask, added with 50 ml of 2M sodium hydroxide aqueous solution, stirred for 0.5h at room temperature, then subjected to standing for 12h at 4 °C. Resulting yellow solid was collected by vacuum filtration and dried to obtain 22.66 g (96% yield) of FuBIG.

CO_2 capture from air using aqueous FuBIG

FuBIG (2.36 g, 10 mmol) was dissolved into 100mL of water and stirred for 12h at room temperature under the condition of sufficiently contacting with air to separate out a yellow solid. The yellow solid was filtered at reduced pressure and dried to obtain yellow powder (3.52 g, 95%), which was $FuBIGH_2(CO_3)(H_2O)_4$.

Single crystal X-ray diffraction

X-ray quality single crystals of $FuBIGH_2(CO_3)(H_2O)_4$ were obtained by preparing an aqueous solution of FuBIG (20 mL, 5 mM) in a 50 mL round bottom flask under ambient air, and let it at room temperature few days. 12423 diffraction points and 5860 independent diffraction points (R_{int} = 0.0204, R_{sigma} = 0.0213) were collected within the range of $2\theta_{max}$ = 134° at T = 100 K using Cu-Kα ray (λ = 1.54184 Å) was used as a light source. The crystal structure was analyzed by XS (Sheldrick, 2008) and refined by SHELXL (Sheldrick, 2015).

pK_a determination by potentiometric titrations

The variable temperature titrations of pK_a of FuBIG were done at the desired temperature using a circulating water bath. The electrode was calibrated by potassium hydrogen phthalate buffer (pH 4.00), mixed phosphate buffer (pH 6.86) and sodium tetraborate buffer (pH 9.18) respectively. A 50 mL ultrapure water solution containing FuBIG (5 mM), HCl (6 mM) and NaCl

background electrolyte (0.2 mM) was titrated with a standard 0.1 M NaOH solution using a 200 μL pipette. The potential readings were recorded 5 minutes after each NaOH addition to allow the solution to equilibrate. The volume and pH value of each titration were recorded. **Table S7** lists the pK_a values obtained in the 15-35 °C range.

Solubility measurements

The solubilities of FuBIG and $FuBIGH_2(CO_3)(H_2O)_4$ under variable temperature were determined by measuring the UV-Vis absorption spectra of the corresponding saturated ultrapure water solutions and comparing with a calibration curve obtained using solutions of $FuBIGH_2Cl_2$ of known concentrations. The $FuBIGH_2Cl_2$ aqueous solutions having the concentrations of 1.346×10^{-5} M, 2.690×10^{-5} M, 3.365×10^{-5} M, 6.730×10^{-5} M and 1.350×10^{-4} M respectively. The absorbance of the $FuBIGH_2Cl_2$ samples was recorded under the maximum absorption wavelength (**Table S3**).

Saturated solutions were prepared by suspending an excess of the crystalline solids in 10 mL H_2O inside reaction tube, and stirred for 24h inside a circulating water bath at different temperatures in the range of 15 to 35 °C. All measurements were run in triplicate. The average solubility values of FuBIG and $FuBIGH_2(CO_3)(H_2O)_4$ are reported respectively in **Table S4** and **S6**.

Determination of K_{sp} of $FuBIGH_2(CO_3)(H_2O)_4$

$$FuBIGH_2(CO_3)(H_2O)_4 \rightleftharpoons FuBIGH_2^{2+} + CO_3^{2-} + 4H_2O \qquad (1)$$

$$HCO_3^- \rightleftharpoons CO_3^{2-} + H^+ \qquad (2)$$

$$FuBIGH_2^{2+} \rightleftharpoons FuBIGH^+ + H^+ \qquad (3)$$

$$FuBIGH^+ \rightleftharpoons FuBIG + H^+ \qquad (4)$$

As the calculation of K_{sp} at 25°C for an example:

K_{sp} is the reaction equilibrium constant of equation 1. The concentration of $FuBIGH_2^{2+}$ and CO_3^{2-} in equilibrium and the activity coefficient ($\gamma_\pm$) at 25°C need be measured and calculated. The concentration of carbonate anion was calculated by equation 2 and mass balance, it was determined to be 0.0001331 M considering the pK_a of HCO_3^- is 10.32 and the pH of the saturated carbonate solution is 8.48.

The concentration of the $FuBIGH_2^{2+}$ cation was determined by taking into account of the measured solubility of $FuBIGH_2(CO_3)(H_2O)_4$ (0.009344 M), the pK_a values of FuBIG in 25 °C (7.57 (equation 3) and 8.71 (equation 4)) and the pH of the saturated solution is 8.48. The concentration of the ligand is: FuBIG 0.003214 M, $FuBIGH^+$ 0.005458 M, $FuBIGH_2^{2+}$ 0.0006715 M.

Ionic strength can be calculated and the value is 0.001609. The activity coefficients ($\gamma_\pm$) were estimated at 0.828 using the Debye-Huckel limiting law (Peiper and Pitzer, 1982; Stefánsson et al., 2013; Huang, 2010). The Values of K_{sp} at other temperature were calculated as the same measurement.

The pK_a of HCO_3^- and value of A in Debye-Huckel limiting law at 15-35 °C were obtained from previous references (Peiper and Pitzer, 1982; Huang, 2010). **Table S5** lists the pH of saturated $FuBIGH_2(CO_3)(H_2O)_4$ solution in the range of 15-35 °C. **Table S6** lists the K_{sp} values obtained in the range of 15-35 °C.

$$I = \frac{1}{2} \times \sum^n cz^2 = \frac{1}{2}(0.0001331 \times 2^2 + 0.0006715 \times 2^2) = 0.001609 \qquad (5)$$

$$\lg \gamma_\pm = -A|Z^+Z^-|\sqrt{I} = -0.5115 \times 4 \times \sqrt{0.001609} = -0.08207 \qquad (6)$$

$$K_{sp} = (\gamma_\pm)^2 \times [FuBIGH_2^{2+}] \times [CO_3^{2-}] = (0.828)^2 \times 0.0006715 \times 0.0001331 = 6.128 \times 10^{-8} \qquad (7)$$

TGA measurements
The TGA was under a nitrogen atmosphere. The sample was ramped at 10 °C/min to 600 °C. For the isothermal measurement, FuBIG carbonate salt was ramped 5 °C/min to 50, 60, 70, 80, 90 °C and 10 °C/min to 100, 110 °C, then held the temperature for 180 min.

DSC measurements
DSC was conducted under a nitrogen atmosphere. The sample was measured in a temperature range of 30-200 °C and temperature ramp of 10 °C/min.

CO_2 absorption and release of FuBIG monitored by ReactIR
The infrared spectrum of FuBIG and FuBIG$H_2(CO_3)(H_2O)_4$ was collected through ReactIR. The peak at 1533 cm^{-1} is the characteristic N-H absorption of FuBIG, while the peak at 1365 cm^{-1} representing the wavenumber of the carbonate salt. The calibration curve was determined by FuBIG aqueous solutions having the concentrations of 0.15890 M and diluted 5, 10, 25 and 50 times respectively. The absorbance of the above concentration FuBIG solutions was measured in ReactIR.
FuBIG (2.83 g, 12 mmol) was added to a 100 mL three-necked flask and dissloved into 40 mL water at 25°C. One neck of flask was inserted into an on-line Infrared Dicomp Probe and fixed with a Teflon adapter. One data is collected every 0.5 min. After the absorbance is stabilized, a CO_2 balloon was inserted into the other mouth of the flask, and continued to stir until FuBIG was completely converted into FuBIG$H_2(CO_3)(H_2O)_4$. Then the mixture was heated to 70 °C, FuBIG$H_2(CO_3)(H_2O)_4$ precipitate released CO_2 and converted to FuBIG aqueous solution again.

Accelerated aging test of FuBIG$H_2(CO_3)(H_2O)_4$
FuBIG$H_2(CO_3)(H_2O)_4$ (0.1850 g, 0.5 mmol) was put on a crystallization dish (24.1800 g). Then placed the crystallization dish into the oven and heated to 110 °C for one week. The weight change of FuBIG$H_2(CO_3)(H_2O)_4$ was measured every 6 hours.

CO_2 separation cycles
FuBIG (9.5 g, 0.04 mol) was dissolved into 100 mL ultrapure water in a 250 mL round bottom flask and marked liquid level. The absorbance of FuBIG at 368 nm was measured to be substituted into the standard equation to calculate the concentration. Then the CO_2 balloon was bubbled through the solution for 1 hour. A yellow precipitate started to form after 5 min. The yellow solid was collected by vacuum filtration. The filtrate was collected and analyzed by UV-Vis spectroscopy to determine the concentration of FuBIG left in solution. The filtrate was saved for the next cycle. The precipitate was placed in a crystallization dish and heated for 4 hours in an oven at 100 °C. The regenerated FuBIG was redissolved into the filtrate saved from the previous cycle and the ultrapure water was added to the mark. And the resulting FuBIG solution was recycled. Overall, ten consecutive cycles had been run.

CO_2 utilization
N-(quinolin-8-yl)benzamide (1.0 g, 4 mmol), diethylamine (827 μL, 8 mmol), DBU (1.79 mL, 12 mmol), cuprous iodide (0.15 g, 0.8 mmol) and manganese dioxide (0.70 g, 8 mmol) were added to a reaction tube, and dissolved with 20 mL DMF. FuBIG$H_2(CO_3)(H_2O)_4$ (2.22 g, 6 mmol) was added to another reaction tube (Luo et al., 2019). The above-mentioned devices were connected via a breather pipe. FuBIG$H_2(CO_3)(H_2O)_4$ was heated at 80 °C to release CO_2. The copper-catalyzed, one-pot three-component oxidative coupling of benzoylaminoquinoline with CO_2 and diethylamine occurred under standard conditions, affording the corresponding *O*-aryl carbamate (2-(quinolin-8-ylcarbamoyl)phenyl diethylcarbamate) in 73% yield (**Figure S24**).

DFT computational
All reported structures were optimized by the density functional theory (DFT) (Kohn and Sham, 1965; Hohenberg and Kohn, 1964) with the B3LYP functional (Vosko et al., 1980; Lee et al., 1988; Becke, 1993) with 6-31G (d, p) basis sets (Petersson et al., 1988, 1991) in the gas phase. Based on the recent studies by Grimme, the empirical dispersion correction was considered to be important in accurate prediction of the reaction free energy (Chakraborty et

al., 2014). Hence, the D3 version of Grimme's dispersion correction with the original D3 damping function was considered in structure optimizations and energy calculations (Grimme et al., 2010). Frequency analysis calculations of optimized structures were performed at the same level of theory to characterize the structures to be minima (no imaginary frequency). Based on the B3LYP-D/6-31G (d, p) optimized geometries, the energy results were further refined by calculating the single point energy at the B3LYP-D/6-311++G (d, p) (Petersson et al., 1988, 1991) level of theory. The bulky solvation effects were simulated by SMD (Marenich et al., 2009) continuum solvent mode at the B3LYP-D/6-311++G (d, p) level of theory, with water (ε = 78.4) and DMSO (ε = 46.8), respectively, according to their corresponding reaction conditions. All the calculations were performed with the Gaussian 09 program (Frisch et al., 2013). The 3D optimized structures were displayed by CYLview visualization program (Legault, 2009).

Zebrafish handling

Adult AB strain zebrafish were fed with live brine shrimp twice daily and dry flake once a day. The culture temperature was controlled by aquaculture facility with a standard 14 h/10 h light/dark photoperiod (Westerfield, 1995). Four to five pairs of zebrafish were set up for nature mating every time. On average, 200–300 embryos were generated. Embryos were maintained at 28 °C in fish water (0.2% Instant Ocean Salt in deionized water, pH 6.9-7.2, conductivity 480-510 μS/cm and hardness 53.7-71.6 mg/L $CaCO_3$). The embryos were washed and staged at 6 h post-fertilization (hpf) and 24 hpf (Kimmel et al., 1995). Zebrafish were housed in Hunter Biotechnology, Inc., which is accredited by the Association for Assessment and Accreditation of Laboratory Animal Care (AAA LAC) International.

Determination of maximum non-lethal concentration (MNLC) and LC_{10}

Zebrafish larvae were treated with FuBIG or PyBIG from 48 to 120 hpf for the acute toxicity, from 4 to 120 hpf for the embryonic toxicity assay (He et al., 2013; Zhu et al., 2014). Mined as those lacking an observable heartbeat under a dissecting stereomicroscope. Seven concentrations were used for each sample. If LC_{10} (10% lethal concentration) and MNLC were not reached, additional testing concentrations up to 2000 μM and down to 0.001 μM were tested. Mortality curves were generated using Origin 8.0 (OriginLab, USA). MNLC and LC_{10} were estimated from this curve.

Identification of target organs

Four concentrations (1/9 MNLC, 1/3 MNLC, MNLC and LC_{10}) were used to identify the toxicity target organs. Zebrafish larvae were treated with samples from 48 to 120 hpf. At the end of treatment, zebrafish from each group were randomly selected for visua observation and image acquisition. Major zebrafish organs and tissues were visually assessed, and toxic target organs were identified based on morphological abnormalities.

After treatment, the heart, brain, eyes, liver, intestine, spine, and behaviours of each fish were observed under the microscope. The occurrence of edema, hemorrhage, and thrombosis were also observed in the animals.

Embryo toxicity

Four concentrations (1/9 MNLC, 1/3 MNLC, MNLC and LC_{10}) were used to identify the embryonic toxicity. Zebrafish larvae were treated with samples from 4 to 120 hpf. At the end of treatment, zebrafish from each group were randomly selected for visual observation and image acquisition. Embryos were daily observed up to 120 h with the dissecting stereomicroscope (SZX7, OLYMPUS, Japan), recording the four apical observations as indicators of lethality: coagulation of fertilized eggs, lack of somite formation, lack of detachment of the tailbud from the yolk sac, and lack of heartbeat. During the exposure period, developmental alterations, teratological parameters, and percentage of hatching were also recorded. Major zebrafish organs and tissues were visually assessed, and toxic target organs were identified based on morphological abnormalities. After treatment, the heart, brain, eyes, liver, intestine, spine, and behaviors of each fish were observed under the microscope. The occurrence of edema, hemorrhage, and thrombosis were also observed in the animals.

Supplemental Figures

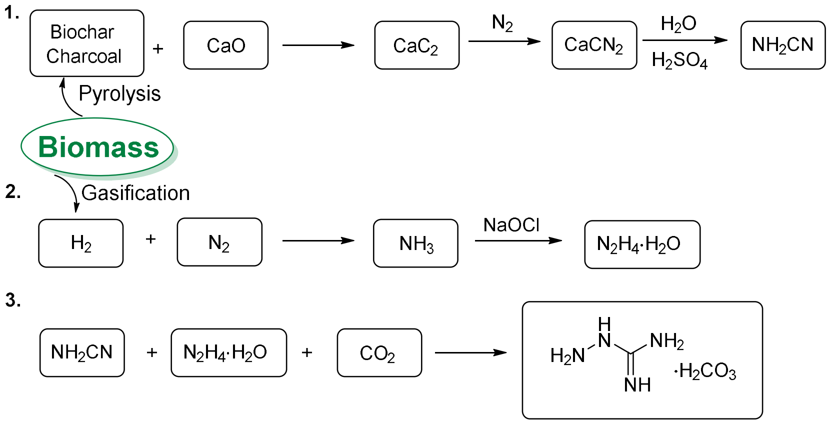

Figure S1. Synthetic routes of biomass derived aminoguanidine. Related to Figure 1.

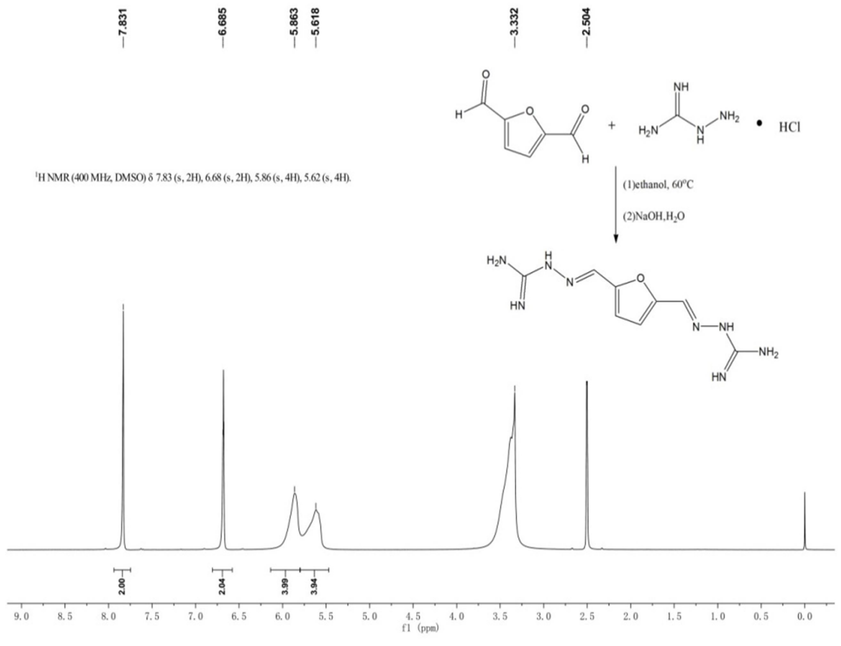

Figure S2. ^{1}H-NMR Spectrum of FuBIG in DMSO-d_6. Related to Figure 2.

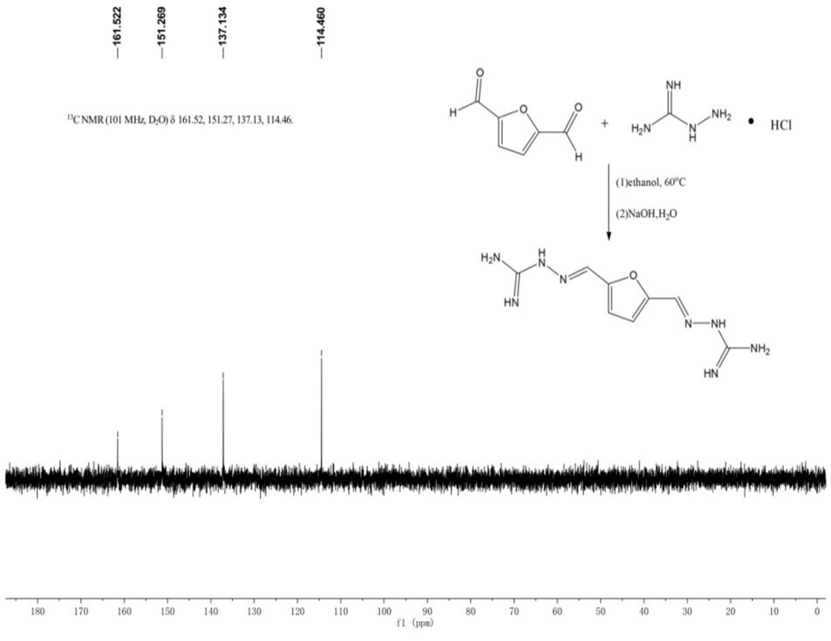

Figure S3. ^{13}C-NMR Spectrum of FuBIG in DMSO-d$_6$. Related to Figure 2.

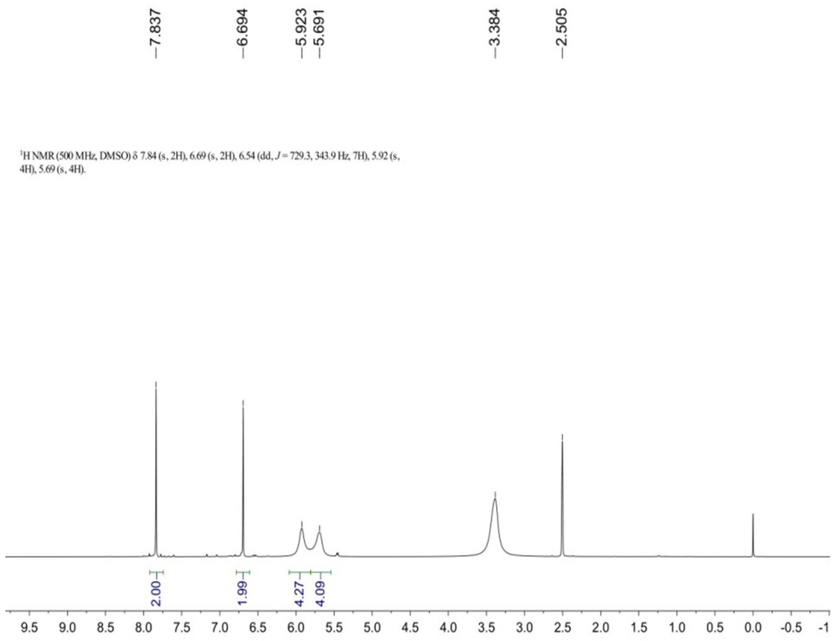

$$\mathrm{FuBIGH_2(CO_3)(H_2O)_4} \underset{\mathrm{H_2O}}{\overset{\mathrm{DMSO}}{\rightleftharpoons}} \mathrm{FuBIG} + \mathrm{CO_2}\uparrow + 5\mathrm{H_2O}$$

Figure S4. ^{1}H-NMR spectrum of $FuBIGH_2(CO_3)(H_2O)_4$ in DMSO-d_6 showing a pattern fully identical with that of FuBIG in DMSO-d_6. Related to Figure 4.

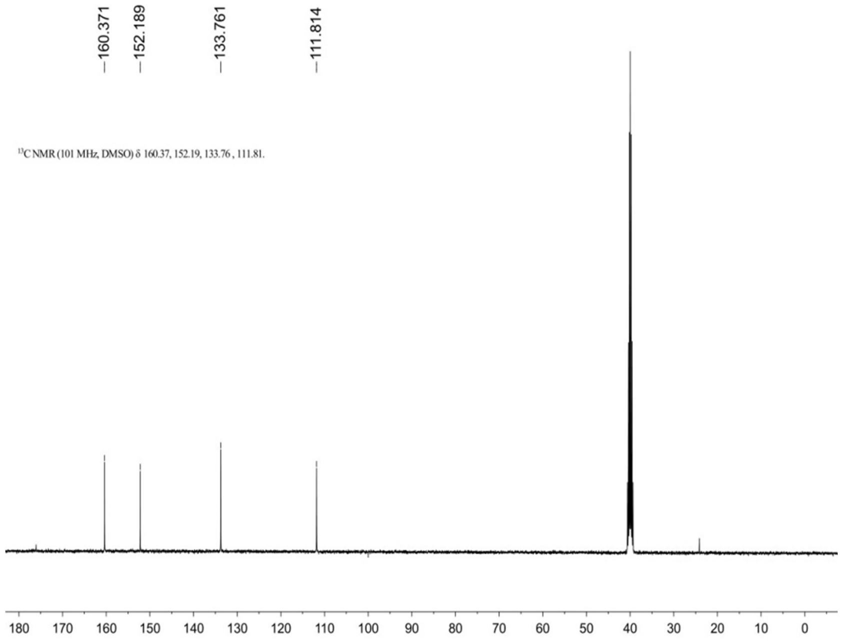

$$FuBIGH_2(CO_3)(H_2O)_4 \underset{H_2O}{\overset{DMSO}{\rightleftharpoons}} FuBIG + CO_2\uparrow + 5H_2O$$

Figure S5. ^{13}C-NMR spectrum of $FuBIGH_2(CO_3)(H_2O)_4$ in DMSO-d_6 showing a pattern fully identical with that of FuBIG in DMSO-d_6. Related to Figure 4.

Elmt	Val.	Min	Max	Elmt	Val.	Min	Max	Elmt	Val.	Min	Max	Use Adduct
H	1	12	12	Si	4	0	0	Se	2	0	0	H
B	3	0	0	P	3	0	0	Br	1	0	0	Na
C	4	8	8	S	2	0	0	Sb	3	0	0	
N	3	0	8	Cl	1	0	0	I	3	0	0	
O	2	0	1	Ni	2	0	0	Ir	3	0	0	
F	1	0	0	Cu	2	0	0	Pt	2	0	0	

Error Margin (ppm): 50
HC Ratio: 0.0 - 1000.0
Max Isotopes: all
MSn Iso RI (%): 75.00

DBE Range: -50.0 - 100.0
Apply N Rule: yes
Isotope RI (%): 1.00
MSn Logic Mode: AND

Electron Ions: both
Use MSn Info: yes
Isotope Res: 10000
Max Results: 100

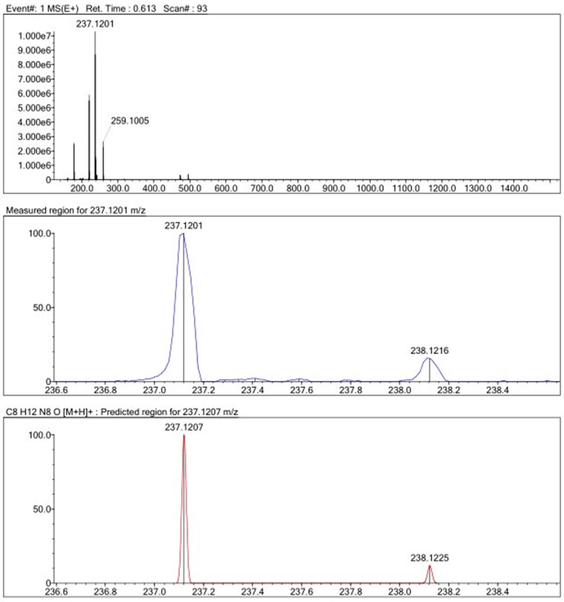

Rank	Score	Formula (M)	Ion	Meas. m/z	Pred. m/z	Df. (mDa)	Df. (ppm)	Iso	DBE
1	50.11	C8 H12 N8 O	[M+H]+	237.1201	237.1207	-0.6	-2.53	52.10	7.0

Figure S6. HRMS spectrum of FuBIG. Related to Figure 2.

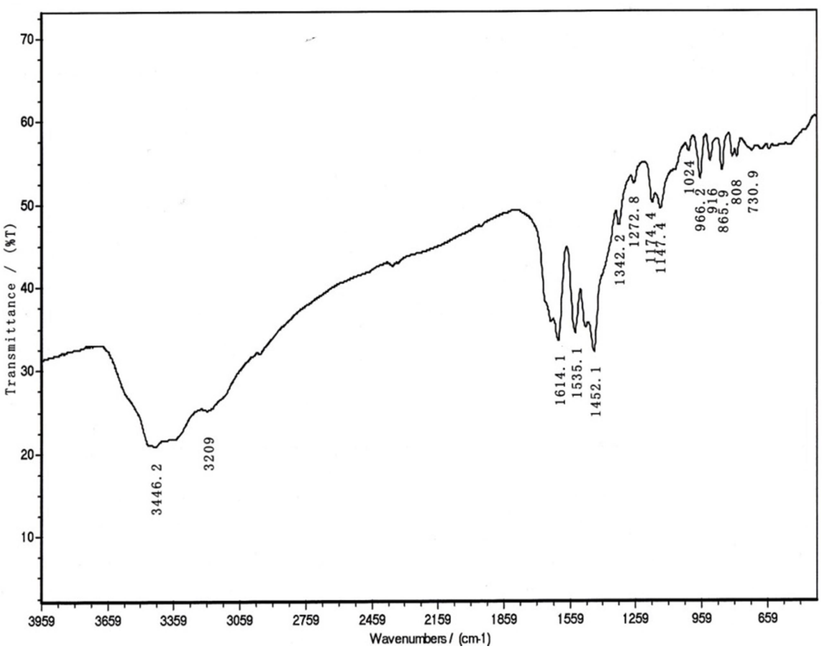

Figure S7. FTIR spectrum of FuBIG. Related to Figure 2.

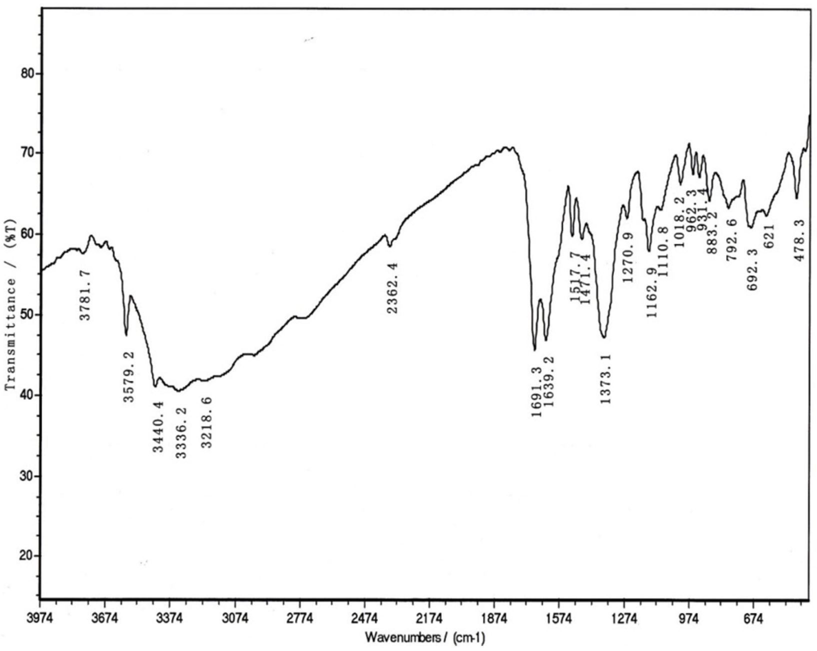

Figure S8. FTIR spectrum of $FuBIGH_2(CO_3)(H_2O)_4$. Related to Figure 2.

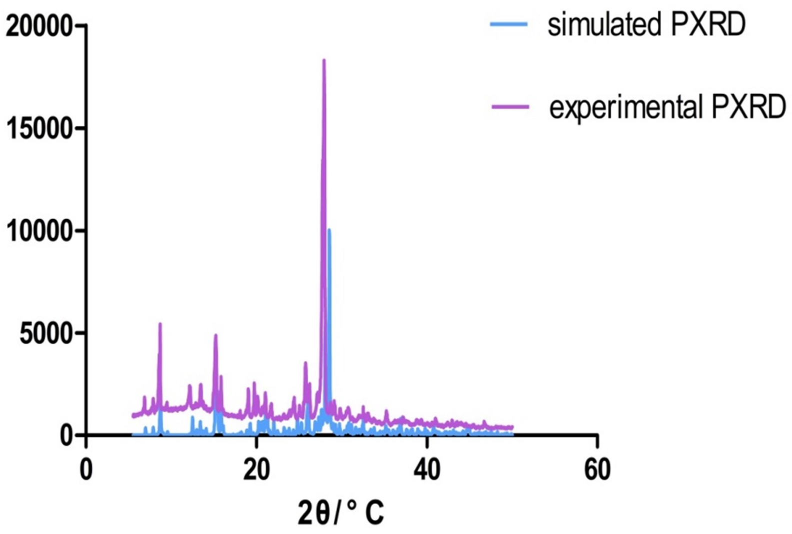

Figure S9. Overlay of the experimental PXRD pattern of the bulk crystalline product (purple) and the simulated PXRD pattern from the single-crystal X-ray data (blue). Related to Figure 2.

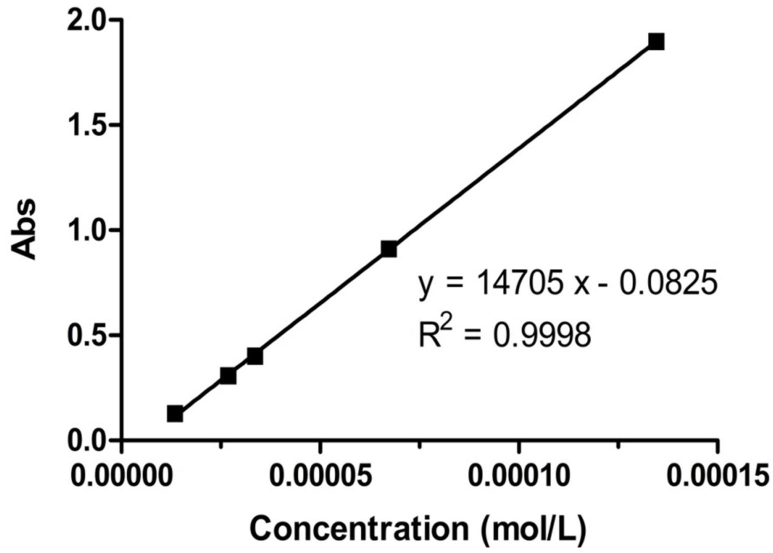

Figure S10. Standard absorption curve of $FuBIGH_2Cl_2$ at 368 nm. Related to Table 1.

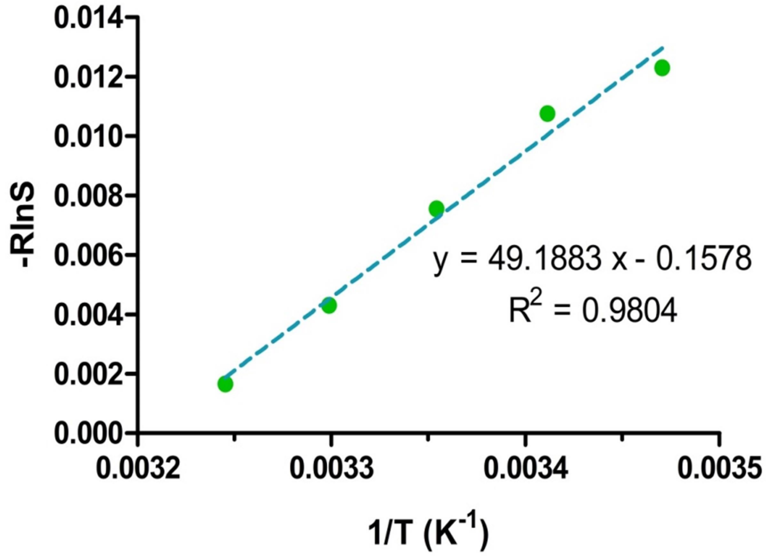

Figure S11. Van't Hoff analysis of FuBIG solubility. Related to Table 1. According to the equation : -Rln K = - ΔH/T + ΔS, the slope of the fitted equation is the value of ΔH in this reaction.

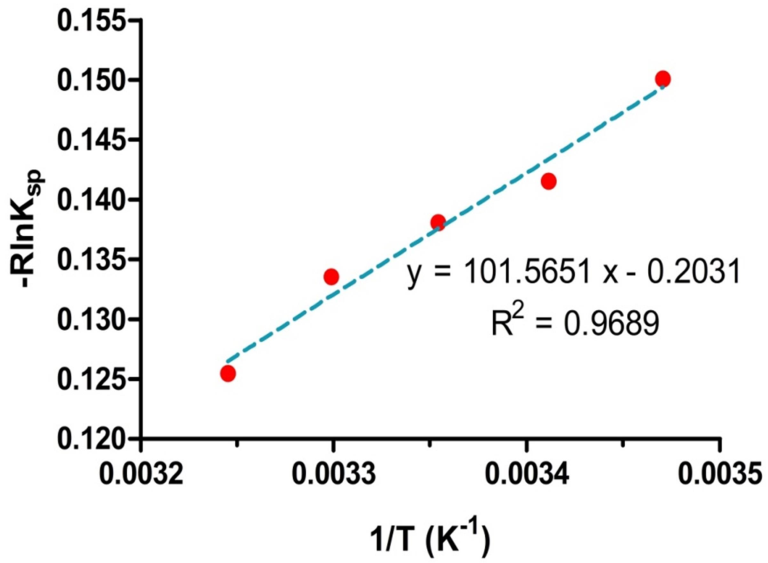

Figure S12. Van't Hoff analysis of solution equilibrium constant of FuBIGH$_2$(CO$_3$)(H$_2$O)$_4$. Related to Table 1. K_{sp} of FuBIGH$_2$(CO$_3$)(H$_2$O)$_4$ at different temperature was calculated respectively to ensure the accuracy of ΔH of FuBIGH$_2$(CO$_3$)(H$_2$O)$_4$ crystallization.

(a)

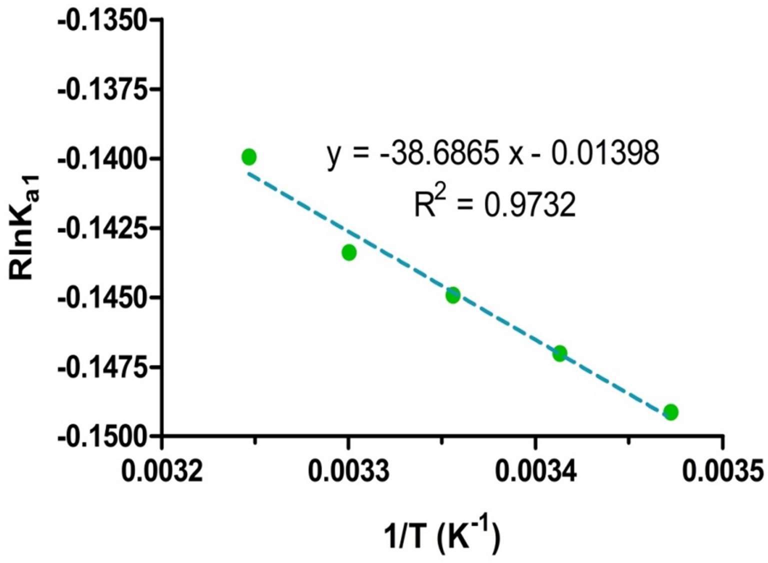

(b)

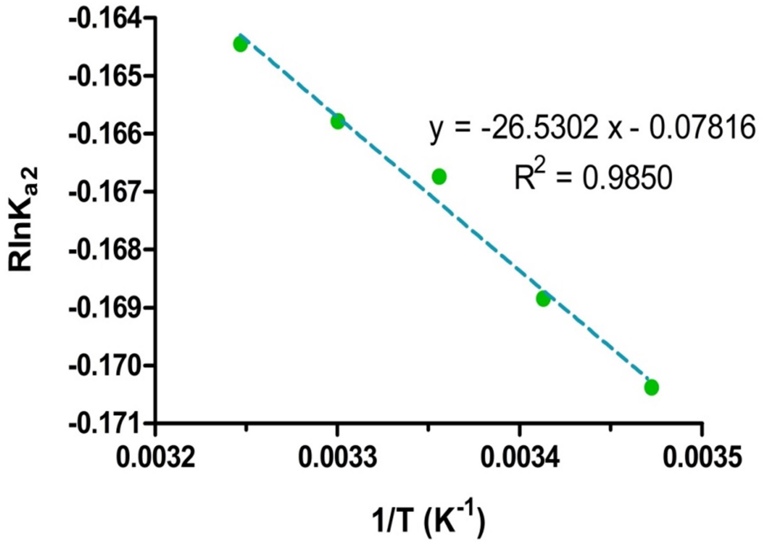

Figure S13. Van't Hoff analysis of $FuBIGH_2^{2+}$ deprotonation. Related to Table 1. (a) Van't Hoff analysis of pK_{a1} of FuBIG. (b) Van't Hoff analysis of pK_{a2} of FuBIG.

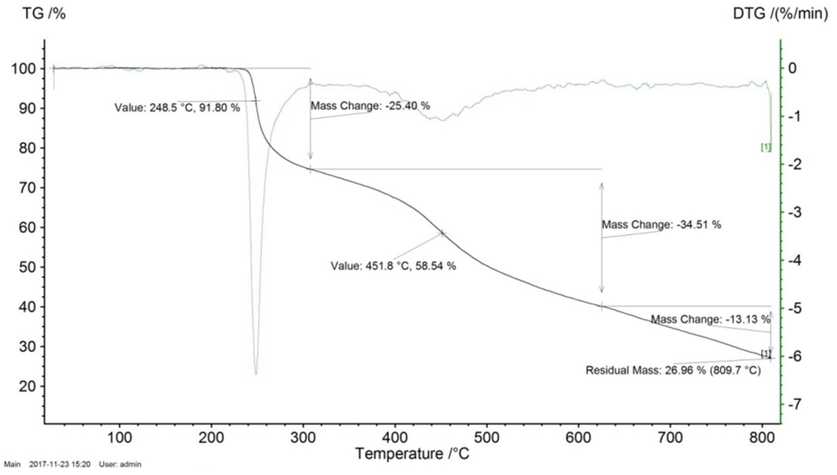

Figure S14. Temperature-ramped TGA plots showing the thermostability of FuBIG. Related to Figure 3.

(a)

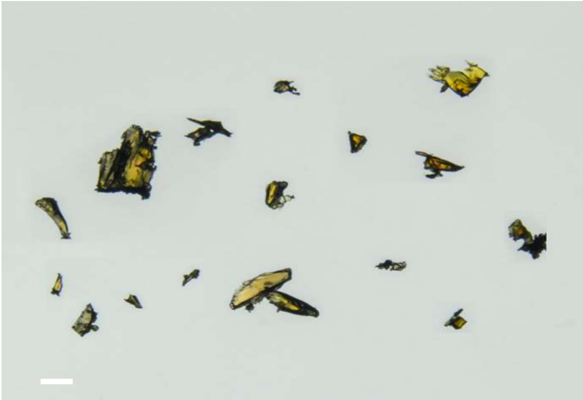

(b)

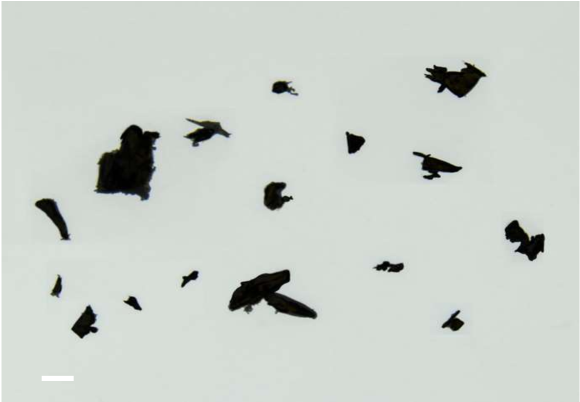

Figure S15. Photos of $FuBIGH_2(CO_3)(H_2O)_4$ crystals under the microscope. Related to Figure 3. (a) $FuBIGH_2(CO_3)(H_2O)_4$ crystals before heating. (b) The crystals of $FuBIGH_2(CO_3)(H_2O)_4$ had been heated in an oven at 120°C for 1h, the crystals transformed to FuBIG and changed their appearance from transparent to opaque. The scale represents 25 μm.

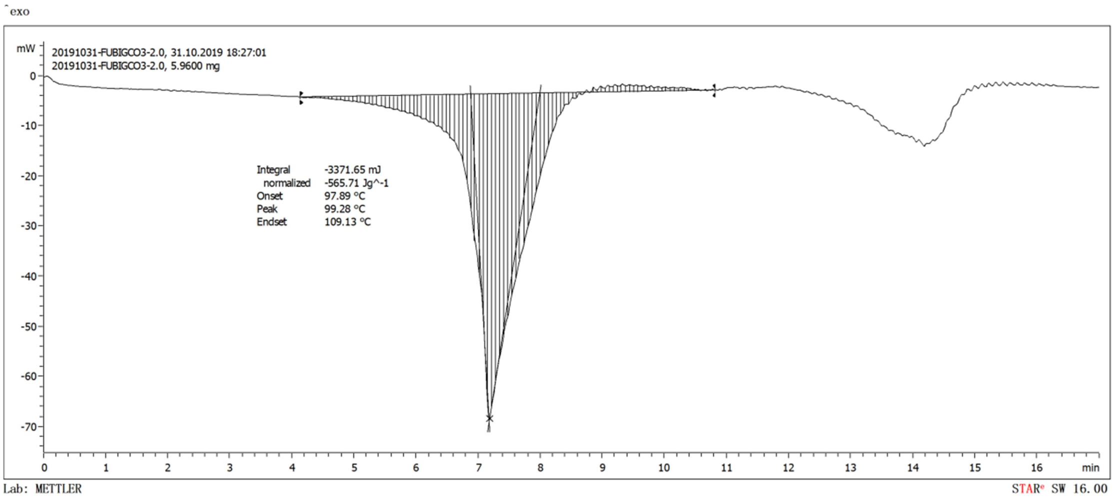

Figure S16. Differential scanning calorimetry (DSC) for CO_2 release of $FuBIGH_2(CO_3)(H_2O)_4$. Related to Table 1.

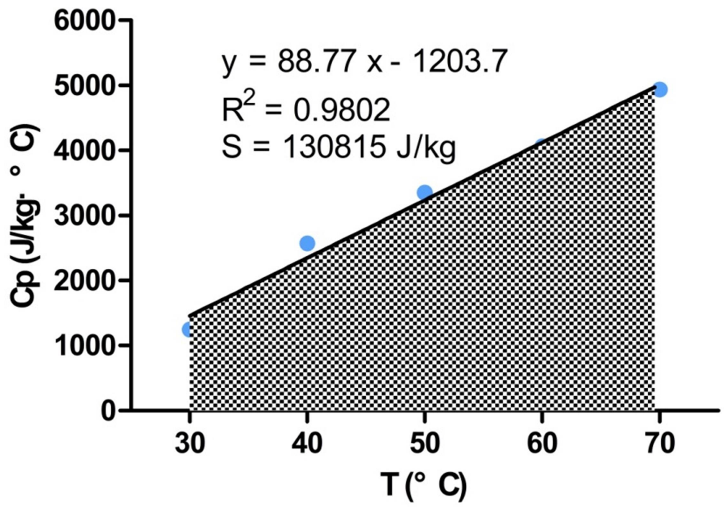

Figure S17. Specific heat capacity of $FuBIGH_2(CO_3)(H_2O)_4$ as a function of temperature, measured by DSC. Related to Table 1. The area bounded by the curve and the abscissa is the enthalpy of the specific heat capacity.

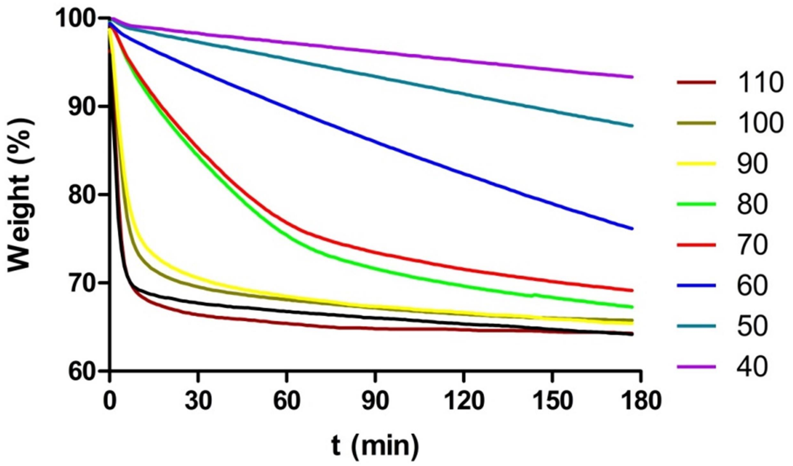

Figure S18. Isothermal TGA data of $FuBIGH_2(CO_3)(H_2O)_4$ at the range of 40-110°C. Related to Figure 3.

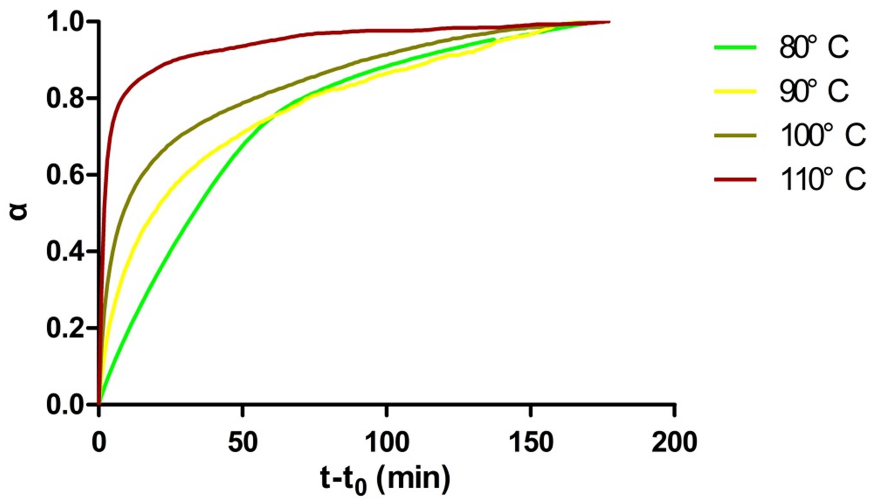

Figure S19. Conversion (α) versus time (t-t_0) plots. Related to Figure 3. t_0 is the time correction that accounts for the induction period, including the time required to heat the sample to the targeted temperature.

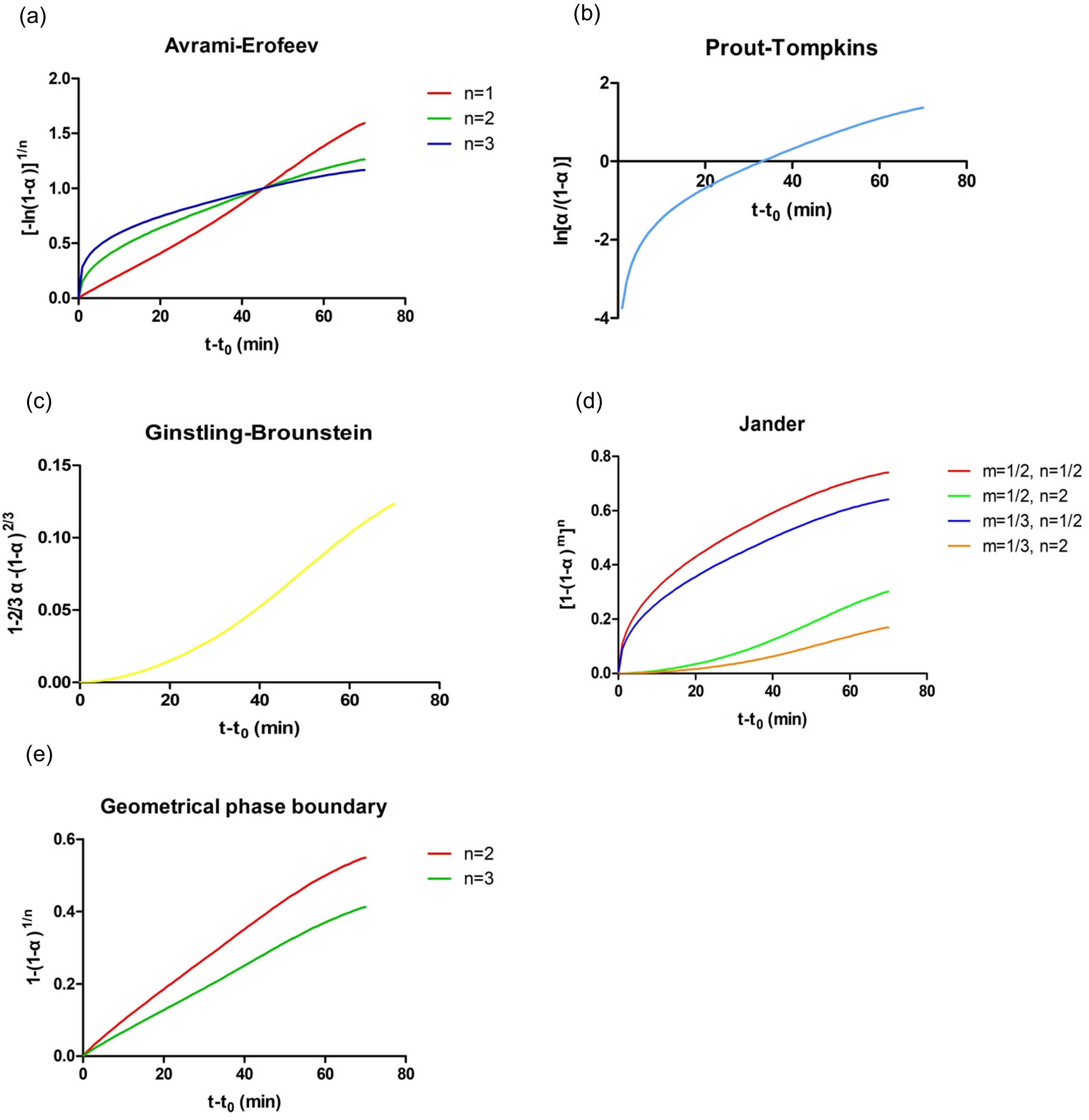

Figure S20. Fitting of the kinetic data to different solid-state kinetic models (showed at 80°C). Related to Figure 3. (a) Avrami-Erofeev model. (b) Prout-Tompkins model. (c) Ginstling-Brounstein model. (d) Jander model. (e) geometrical phase boundary model.

(a)

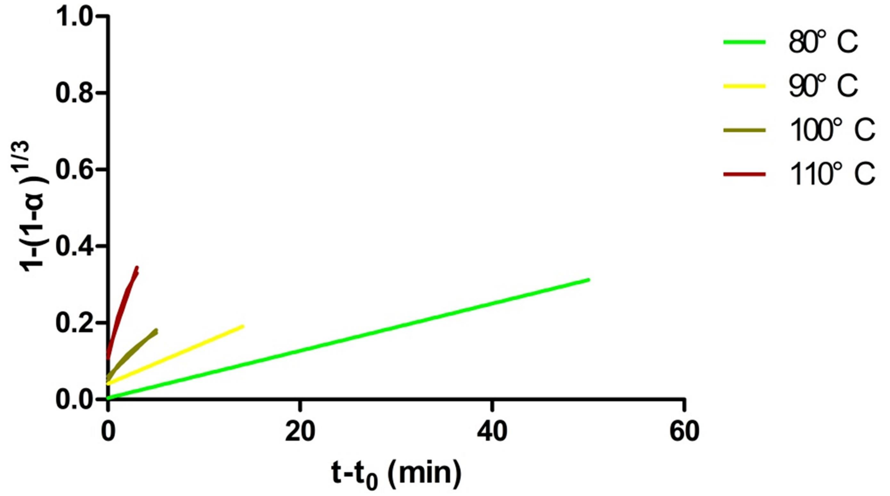

(b)

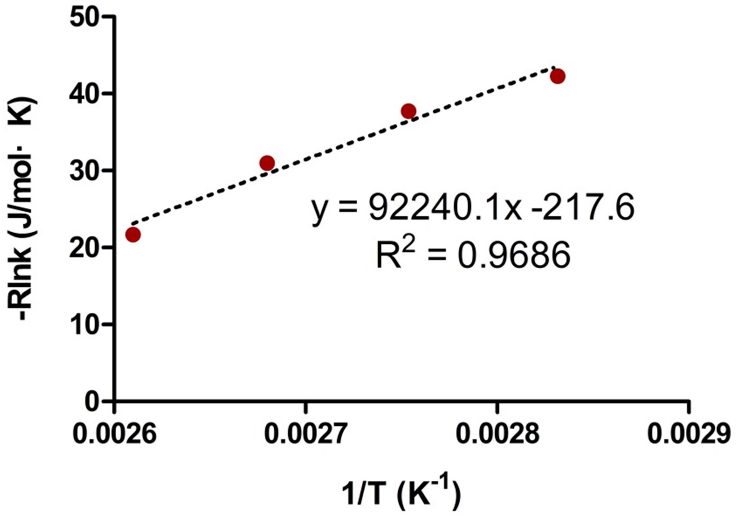

Figure S21. Physicochemical modeling of FuBIG carbonate salt heat release. Related to Figure 3. (a) Fitting of the kinetic data to geometrical phase boundary model. 80°C: y = 0.0062x + 0.0039, R^2 = 0.9998; 90°C: y = 0.0107x + 0.0411, R^2 = 0.9775; 100°C: y = 0.0241x + 0.0608, R^2 = 0.9743; 110°C: y = 0.0736x +0.1240, R^2 = 0.9652. (b) Activation barrier was obtained from Arrhenius analysis of the rate constants (k) under different temperatures.

(a)

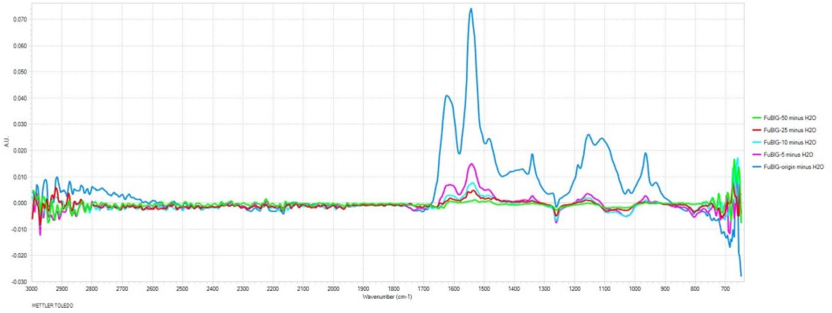

(b)

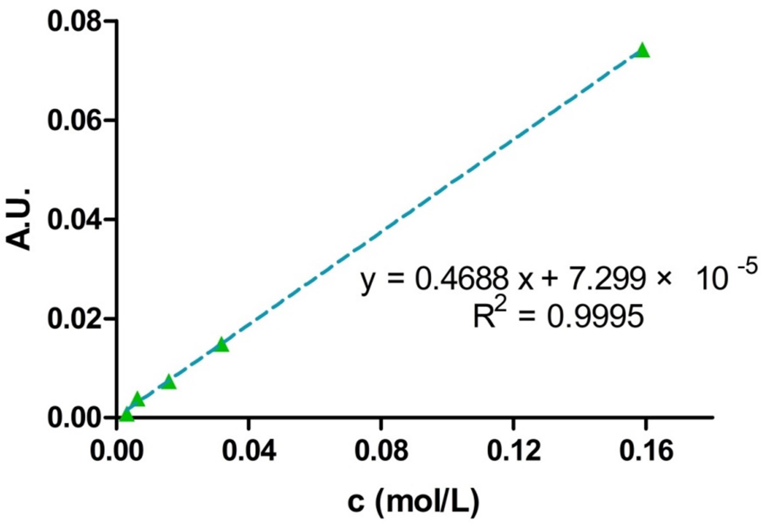

Figure S22. The mathematical relationship between concentration and absorption unit of FuBIG water solution. Related to Figure 3. (a) Absorption of different concentrations of FuBIG in ReactIR. (b) Standard absorption curve of FuBIG concentrate and absorption unit in ReactIR.

(a)

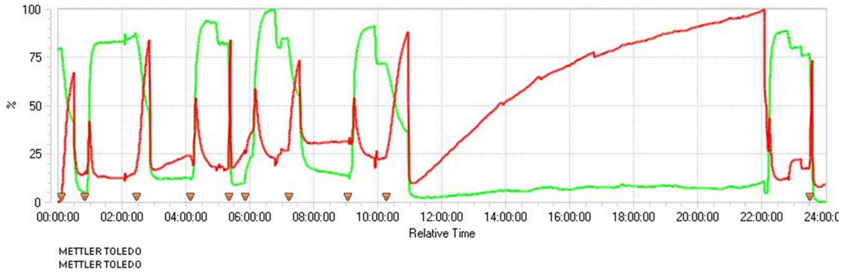

(b)

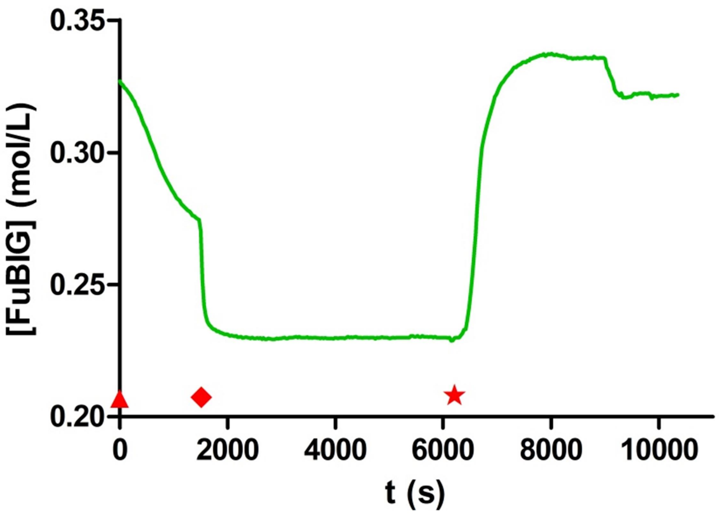

Figure S23. The process of CO_2 absorption-release cycle monitored by React IR. Related to Figure 3. (a) The total process of CO_2 absorption-release of FuBIG. (b) The kinetic profiles of CO_2 absorption were picked between 2:26:55 and 2:48:55. Tiangle: representing the time point for the adding of CO_2 into the solvent. Rhombus: representing the time point for complete conversion of FuBIG to $FuBIGH_2(CO_3)(H_2O)_4$. Five-pointed star: representing the time point for heating.

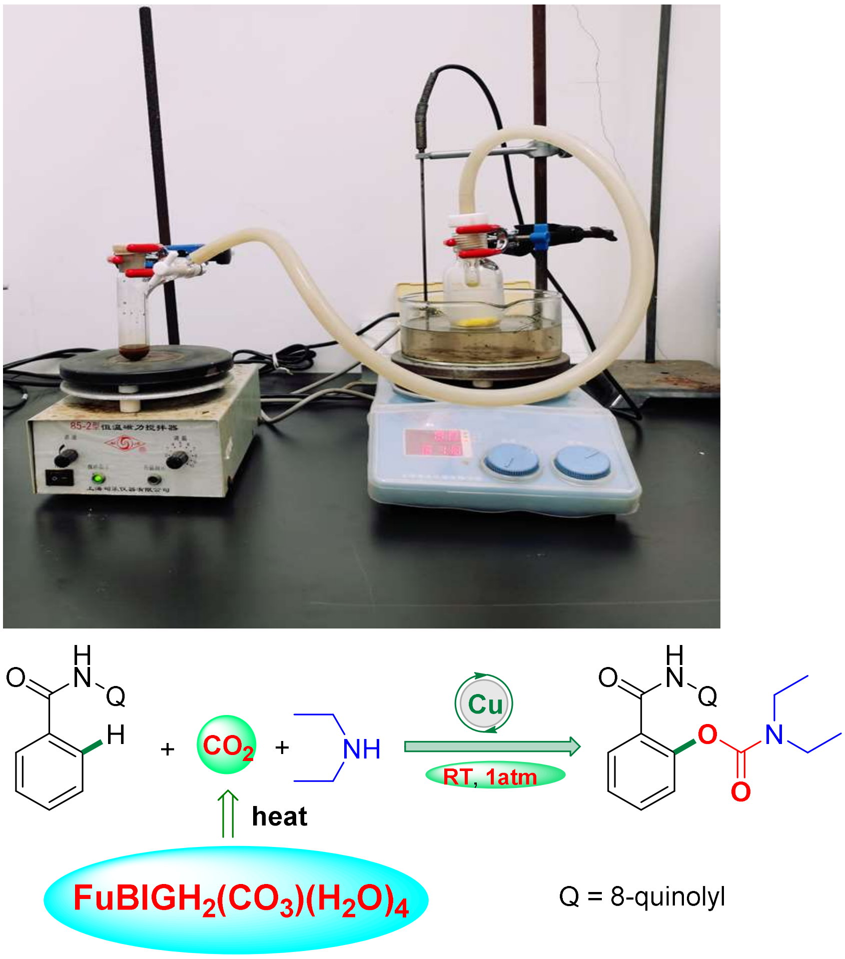

Figure S24. Synthetic utility of CO_2 captured by FuBIG in the copper-catalyzed C−H carbamoyloxylation reaction. Related to Figure 3. CO_2 released from $FuBIGH_2(CO_3)(H_2O)_4$ can be utilized in our previous published transformations. (*Org. Lett. 2019, 21, 2013−2018.*)

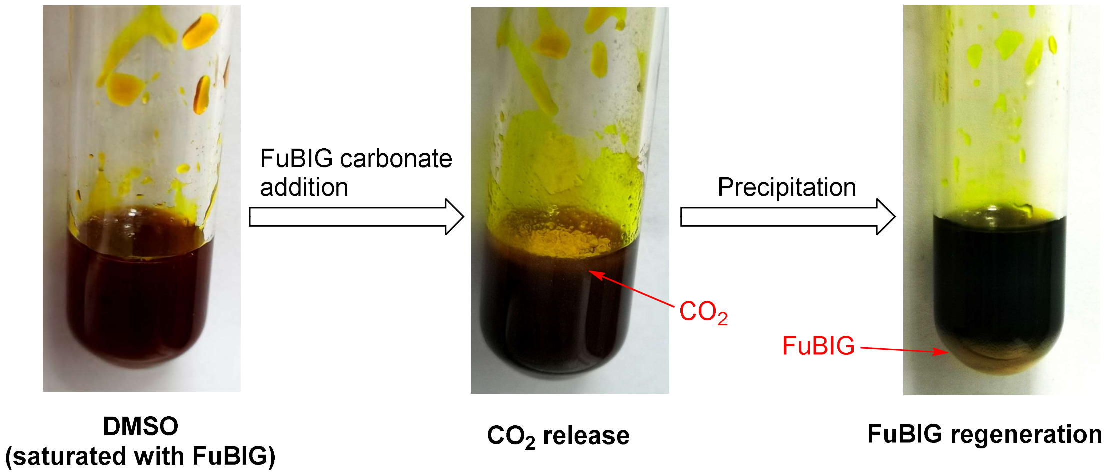

Figure S25. Practical process of FuBIG regeneration with DMSO. Related to Figure 4.

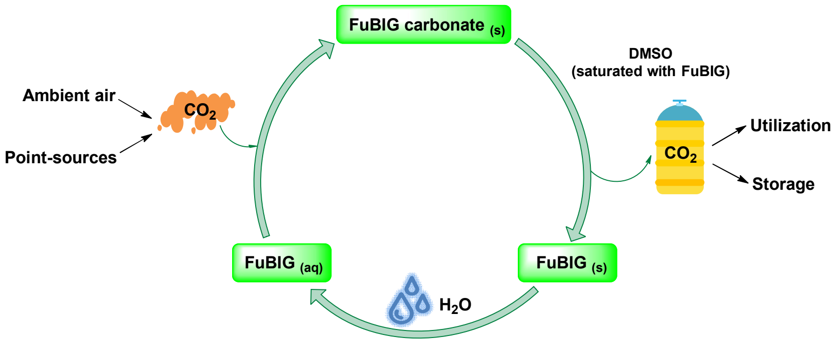

Figure S26. A low-energy protocol for CO_2 capture/release and sorbent regeneration with minimum DMSO consumption. Related to Figure 4. DMSO solution saturated with FuBIG could still promote the release of CO2 from FuBIG carbonate, and lead to the regeneration of FuBIG.

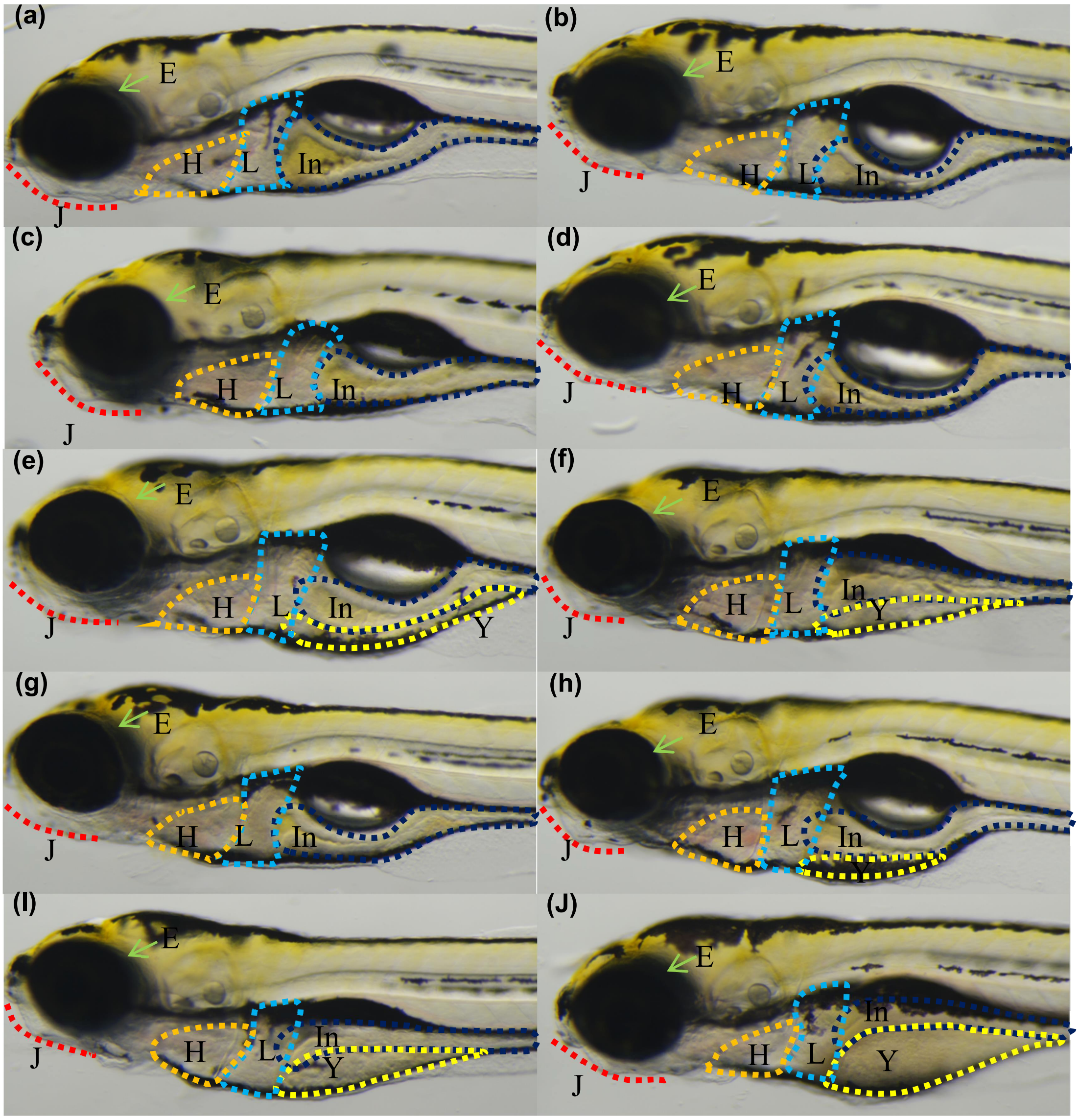

Figure S27. Toxicity of FuBIG and PyBIG in zebrafish testing. Related to Figure 5. (a) Untreated normal control. (b) Zebrafish were treated with 1% DMSO as solvent control. (c) FuBIG 1/9 MNLC. (d) FuBIG 1/3 MNLC. (e) FuBIG MNLC. (f) FuBIG LC_{10}. (g) PyBIG 1/9 MNLC. (h) PyBIG 1/3 MNLC. (I) PyBIG MNLC. (J) PyBIG LC_{10}. H = heart, J = Jaw, In = Intestinal tract, L = Liver, E = eye, Y = Yolk Sac.

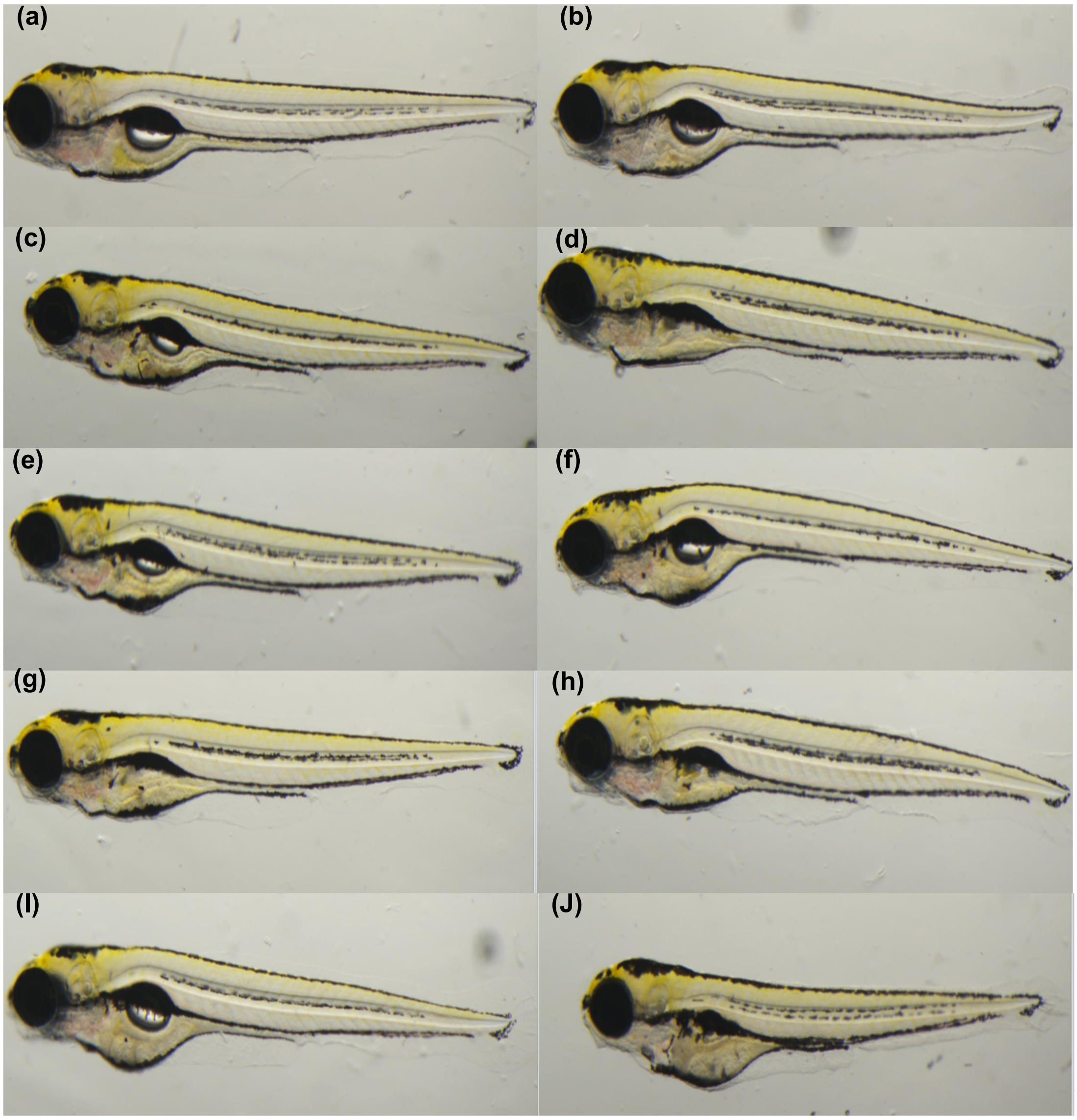

Figure S28. Toxicity of FuBIG and PyBIG in zebrafish embryo testing. Related to Figure 5. (a) Untreated normal control. (b) Zebrafish embryos were treated with 1% DMSO as solvent control. (c) FuBIG 1/9 MNLC. (d) FuBIG 1/3 MNLC. (e) FuBIG MNLC. (f) FuBIG LC_{10}. (g) PyBIG 1/9 MNLC. (h) PyBIG 1/3 MNLC. (I) PyBIG MNLC. (J) PyBIG LC_{10}.

Supplemental Tables

Table S1. Elemental analysis of FuBIG$H_2(CO_3)(H_2O)_4$. Related to Figure 2.

	C [%]	H [%]	N [%]	C/N	C/H
measured	28.50	5.58	29.06	0.9809	5.1096
theoretical	29.19	5.99	30.26	0.9646	4.8731

Table S2. Crystal test parameters of FuBIGH$_2$(CO$_3$)(H$_2$O)$_4$. Related to Figure 2.

Chemical Formula	$C_9H_{22}N_8O_8$
Formula Weight	370.34
Crystal Size (Mm^3)	0.5×0.3×0.2
Crystal System	triclinic
Space Group	P-1
a (Å)	11.1919 (4)
b (Å)	12.7930 (5)
c (Å)	14.1784 (4)
α (deg)	97.547 (3)
β (deg)	111.174 (3)
γ (deg)	112.709 (4)
volume (Å^3)	1657.46 (11)
Z	4
density (g/cm^3)	1.484
2θ range	7.044–134.138
F (000)	787.0
index ranges	-13 ≤ h ≤ 13<br>-15 ≤ k ≤ 15<br>-11 ≤ l ≤ 16
no. of reflns	11953
no. of unique reflns	5860
no. of params	501
R_{all}, R_{obs}	0.0352, 0.0336
$wR_{2,all}$, $wR_{2,obs}$	0.0892, 0.0880
goodness-of-fit on F^2	1.054

$R_1 = \Sigma||F_o| - |F_c||/\Sigma|F_o|$, $wR^2=[\Sigma[w(F_o^2 - F_c^2)^2]/\Sigma w(F_o^2)^2]^{1/2}$,

$w=1/[\sigma^2(F_o)^2 + (aP^2) + bP]$, where $P=[(F_o^2) + 2F_c^2]/3$.

Table S3. Absorption data for $FuBIGH_2Cl_2$ in different concentrations. Related to Table 1.

Concentration (mol/L)	Absorption
0.00001346	0.1276
0.00002690	0.3067
0.00003365	0.4016
0.00006730	0.9116
0.0001346	1.8974

Table S4. Solubility data for FuBIG at 15-35°C. Related to Table 1.

T (°C)	Solubility (mol/L)
15	0.2277
20	0.2739
25	0.4029
30	0.5953
35	0.8192

Table S5. pH data of saturated solution of $FuBIGH_2(CO_3)(H_2O)_4$ at 15-35°C. Relate to Table 1.

T (°C)	pH
15	8.82
20	8.68
25	8.48
30	8.31
35	8.17

Table S6. Solubility data and calculated value for $FuBIGH_2(CO_3)(H_2O)_4$ at 15-35°C. Relate to Table 1.

T (°C)	Solubility (mol/L)	K_{sp} (×10^{-8})
15	0.003899	1.446
20	0.007068	4.042
25	0.009344	6.128
30	0.01318	10.55
35	0.02713	27.92

Table S7. pK_a data for FuBIG at 15-35°C. Relate to Table 1.

T (°C)	pK_{a1}	pK_{a2}
15	7.79	8.90
20	7.68	8.82
25	7.57	8.71
30	7.49	8.66
35	7.31	8.59

Table S8. Absorption data for different concentrations FuBIG in ReactIR. Related to Figure 3.

Concentration (mol/L)	Absorption
0.1589	0.07423
0.03178	0.01490
0.01589	0.007342
0.006356	0.003933
0.003178	0.00082

Table S9. Absorption intensity data monitored at 1533 cm^{-1} (N—H) using React IR when FuBIG carbonate salt being added into DMSO and H_2O respectively. Relate to Figure 4.

Time	A.U. at 1533cm^{-1} in DMSO	A.U. at 1533cm^{-1} in H_2O
00:00:12	0.015584	0.025983
00:00:27	0.015662	0.026056
00:00:42	0.015882	0.025833
00:00:57	0.015789	0.025448
00:01:11	0.015807	0.025254
00:01:27	0.015575	0.025444
00:01:42	0.016172	0.025258
00:01:56	0.015863	0.025088
00:02:11	0.015899	0.025232
00:02:27	0.015468	0.024927
00:02:42	0.015371	0.02474
00:02:57	0.015817	0.02494
00:03:12	0.015371	0.024817
00:03:27	0.01594	0.024991
00:03:42	0.015832	0.024574
00:03:57	0.015571	0.024649
00:04:12	0.016137	0.024753
00:04:26	0.015312	0.024912
00:04:42	0.015361	0.024799
00:04:57	0.015571	0.02493
00:05:12	0.01539	0.024532
00:05:27	0.015058	0.025246
00:05:41	0.015251	0.024929
00:05:57	0.015309	0.024906
00:06:11	0.015094	0.025181
00:06:27	0.015784	0.025324
00:06:41	0.01527	0.025004
00:06:57	0.015581	0.024744
00:07:12	0.015252	0.025361
00:07:27	0.015331	0.023964
00:07:41	0.015134	0.022766
00:07:57	0.021827	0.022361
00:08:12	0.053243	0.021889
00:08:27	0.062639	0.022015
00:08:42	0.065978	0.02145
00:08:56	0.068484	0.021327
00:09:12	0.069669	0.021291
00:09:27	0.07039	0.020931
00:09:42	0.070876	0.020613
00:09:56	0.071583	0.02072

00:10:12	0.072118	0.020691
00:10:27	0.072285	0.020332
00:10:41	0.072672	0.02057
00:10:56	0.072637	0.020668
00:11:12	0.072977	0.020292
00:11:27	0.072714	0.020487
00:11:42	0.072604	0.020336
00:11:57	0.072801	0.020162
00:12:11	0.072886	0.020259
00:12:27	0.073042	0.020169
00:12:41	0.07315	0.019988
00:12:57	0.072603	0.019994
00:13:11	0.073017	0.020357
00:13:27	0.07296	0.019976
00:13:42	0.072799	0.020263
00:13:57	0.073242	0.020133
00:14:12	0.073223	0.020115
00:14:27	0.073186	0.019913
00:14:42	0.073206	0.020044
00:14:57	0.073004	0.019948
00:15:11	0.073203	0.019992
00:15:26	0.073339	0.019996
00:15:42	0.07338	0.019932
00:15:57	0.07317	0.019884
00:16:12	0.07324	0.020251
00:16:27	0.073177	0.019679
00:16:42	0.073341	0.019492
00:16:57	0.073435	0.019708
00:17:11	0.073564	0.019867
00:17:26	0.073634	0.020031
00:17:42	0.073688	0.01999
00:17:57	0.073644	0.019939
00:18:12	0.073695	0.019911
00:18:27	0.073571	0.019986
00:18:41	0.073489	0.019676
00:18:57	0.073555	0.019782
00:19:12	0.073759	0.019742
00:19:27	0.073833	0.019545
00:19:41	0.07413	0.019581
00:19:57	0.073995	0.019713
00:20:12	0.073727	0.019597
00:20:27	0.073561	0.019759
00:20:42	0.073851	0.01975
00:20:56	0.073646	0.019978

00:21:12	0.073997	0.019833
00:21:27	0.073841	0.019925
00:21:41	0.074038	0.019842
00:21:56	0.073841	0.020232
00:22:12	0.074188	0.019747
00:22:26	0.073814	0.020204
00:22:41	0.074055	0.019937
00:22:56	0.073699	0.019965
00:23:12	0.073961	0.019619
00:23:27	0.074076	0.019829
00:23:42	0.073911	0.019745
00:23:56	0.073793	0.019707
00:24:11	0.073845	0.019662
00:24:27	0.073832	0.019742
00:24:42	0.074056	0.019888
00:24:57	0.074118	0.019721

Table S10. Acute toxicity experiment of zebrafish treated with FuBIG and PyBIG. Related to Figure 5.

	Concentration (μM)	Death number	Mortality (%)
Normal	-	0	0
1 % DMSO	-	0	0
FuBIG	4.2	0	0
	21.2	0	0
	42.4	0	0
	75.8	21	70.0
	79.7	23	76.7
	127.1	30	100
	169.5	30	100
PyBIG	3.4	0	0
	17.1	0	0
	30.8	13	43.3
	32.5	16	53.3
	34.2	20	66.7
	68.5	30	100
	102.7	30	100
	137.0	30	100

Table S11. Embryo toxicity experiment of zebrafish embryos treated with FuBIG and PyBIG. Related to Figure 5.

	Concentration (μM)	Death number	Mortality (%)
Normal	-	0	0
1 % DMSO	-	0	0
FuBIG	21.2	0	0
	42.4	0	0
	84.7	10	33.3
	127.1	19	63.3
	169.5	30	100
	211.9	30	100
PyBIG	17.1	0	0
	34.2	8	26.7
	68.5	29	96.7
	102.7	30	100
	137.0	30	100
	171.2	30	100

References

Becke, A.D. (1993). Density-functional thermochemistry. III. The role of exact exchange. J. Chem. Phys. *98*, 5648–5652.

Chakraborty, S., Lagaditis, P.O., Förster, M., Bielinski, E.A., Hazari, N., Holthausen, M.C., Jones, W.D. and Schneider, S. (2014). Well-defined iron catalysts for the acceptorless reversible dehydrogenation-hydrogenation of alcohols and ketones. ACS Catal. *4*, 3994–4003.

Hohenberg, P. and Kohn, W. (1964). Inhomogeneous electron gas. Physical Review *136*, B864–B871.

Frisch, M.J., Trucks, G.W., Schlegel, H.B., Scuseria, G.E., Robb, M.A., Cheeseman, J.R., Scalmani, G., Barone. V., Mennucci, B., Petersson, G.A., et al. (2013). Gaussian 09, Revision D.01 (Gaussian, Inc., Wallingford CT).

Grimme, S., Antony, J., Ehrlich, S. and Krieg, H. (2010). A consistent and accurate *ab initio* parametrization of density functional dispersion correction (DFT-D) for the 94 elements H-Pu. J. Chem. Phys. *132*, 154104.

He, J.H., Guo, S.Y., Zhu, F., Zhu, J.J., Chen, Y.X., Huang, C.J., Gao, J.M., Dong, Q.X., Xuan, Y.X. and Li, C.Q. (2013). A zebrafish phenotypic assay for assessing drug-induced hepatotoxicity. J. Pharmacol. Toxicol. Methods. *67*, 25–32.

Huang, Z. (2010). Introduction of electrolyte solution theory (Science Press).

Kimmel, C.B., Ballard, W.W., Kimmel, S.R., Ullmann, B. and Schilling, T.F. (1995). Stages of embryonic development of the zebrafish. Developmental dynamics *203*, 253–310.

Kohn, W. and Sham, L.J. (1965). Self-consistent equations including exchange and correlation effects. Physical Review *140*, A1133–A1138.

Lee, C., Yang, W. and Parr, R.G. (1988). Development of the Colle-Salvetti correlation-energy formula into a functional of the electron density. Phys Rev B Condens Matter *37*, 785–789.

Legault, C.Y. (2009). CYLview, 1.0b (Université de Sherbrooke). http://www.cylview.org.

Luo, X., Song, X., Xiong, W., Li, J., Li, M., Zhu, Z., Wei, S., Chan, A.S.C. and Zou, Y. (2019). Copper-catalyzed C–H carbamoyloxylation of aryl carboxamides with CO_2 and amines at ambient conditions. Org. Lett. *21*, 2013–2018.

Marenich, A.V., Cramer, C.J. and Truhlar, D.G. (2009). Universal solvation model based on solute electron density and on a continuum model of the solvent defined by the bulk dielectric constant and atomic surface tensions. J. Phys. Chem. B. *113*, 6378–6396.

Peiper, J.C. and Pitzer, K.S. (1982). Thermodynamics of aqueous carbonate solutions including mixtures of sodium carbonate, bicarbonate, and chloride. J. Chem. Thermodynamics. *14*, 613–638.

Petersson, G.A. and Al-Laham, M.A. (1991). A complete basis set model chemistry. II. Open-shell systems and the total energies of the first-row atoms. J. Chem. Phys. *94*, 6081–6090.

Petersson, G.A., Bennett, A., Tensfeldt, T.G., Al-Laham, M.A., Shirley, W.A. and Mantzaris, J. (1988). A complete basis set model chemistry. I. The total energies of closed-shell atoms and hydrides of the first-row elements. J. Chem. Phys. *89*, 2193–2218.

Stefánsson, A., Bénézeth, P. and Schott, J. (2013). Carbonic acid ionization and the stability of sodium bicarbonate and carbonate ion pairs to 200 C – A potentiometric and spectrophotometric study. Geochimica. et. Cosmochimica. Acta. *120*, 600–611.

Vosko, S.H., Wilk, L. and Nusair, M. (1980). Accurate spin-dependent electron liquid correlation energies for local spin density calculations: a critical analysis. Canadian Journal of Physics *58*, 1200–1211.

Westerfield M. (1995). The Zebrafish Book. A Guide for the Laboratory Use of Zebrafish (Danio rerio) (University of Oregon Press).

Zhu, J.J., Xu, Y.Q., He, J.H., Yu, H.P., Huang, C.J., Gao, J.M., Dong, Q.X., Xuan, Y.X. and Li, C.Q. (2014). Human cardiotoxic drugs delivered by soaking and microinjection induce cardiovascular toxicity in zebrafish. J. Appl. Toxicol. *34*, 139–148.
